# Synthesis and Use of Stable Isotope Enriched Retinals in the Field of Vitamin A

**DOI:** 10.3390/molecules15031825

**Published:** 2010-03-15

**Authors:** Prativa B.S. Dawadi and Johan Lugtenburg

**Affiliations:** Leiden Institute of Chemistry, Leiden University, P.O. Box 9502, 2300 RA, Leiden, The Netherlands; E-Mail: p.b.s.dawadi@gmail.com (P.B.S.D.)

**Keywords:** wittig reaction, nitrile chemistry, retinals, deuterium, ^13^C-incorporation

## Abstract

The role of vitamin A and its metabolites in the life processes starting with the historical background and its up to date information is discussed in the introduction. Also the role of 11*Z*-retinal in vision and retinoic acid in the biological processes is elucidated. The essential role of isotopically enriched systems in the progress of vision research, nutrition research *etc.* is discussed. In part B industrial commercial syntheses of vitamin A by the two leading companies Hoffmann-La Roche (now DSM) and BASF are discussed. The knowledge obtained *via* these pioneering syntheses has been essential for the further synthetic efforts in vitamin A field by other scientific groups. The rest of the paper is devoted to the synthetic efforts of the Leiden group that gives an access to the preparation of site directed high level isotope enrichment in retinals. First the synthesis of the retinals with deuterium incorporation in the conjugated side chain is reviewed. Then, ^13^C-labeled retinals are discussed. This is followed by the discussion of a convergent synthetic scheme that allows a rational access to prepare any isotopomer of retinals. The schemes that provide access to prepare any possible isotope enriched chemically modified systems are discussed. Finally, nor-retinals and bridged retinals that give access to a whole (as yet incomplete) library of possible isotopomers are reviewed.

## Contents

Part A Introduction

Part B Technical Syntheses

1. Hoffmann-La Roche (now DSM)

2. BASF

Part C Site-Directed Highly Stable Isotope Enriched Retinals

1. Deuterium Labeled Retinals

1.1. Incorporation of deuterium at positions 18 and 19 of retinal 1: Preparation of 11*Z*- [18-D_3_]-retinal and 11*Z*-[19-D_3_]-retinal 

1.2. Incorporation of deuterium at positions 10, 11, 12, 14 and 20 of retinal 1: Preparation of all-E [10-D]-retinal, all-E [11-D]-retinal, all-E [12-D]-retinal, all-E [11,12-D_2_]-retinal, all-E [10,11-D_2_]-retinal, all-E [14,20,20,20-D_4_]-retinal 

1.3. Incorporation of deuterium at positions 14, 15 and 20 of retinal 1: Preparation of all-E [14-D]-retinal, all-E [15-D]-retinal, all-E [14,15-D_2_]-retinal and all-E [20,20,20-D_3_]-retinal

2. ^13^C-Labeled Retinals

2.1. Incorporation of ^13^C at positions 14 and 15 of retinal 1: Preparation of [14-^13^C]-retinal, [15-^13^C]-retinal and [14,15-^13^C_2_]-retinal

2.2. Incorporation of ^13^C in the six membered ring of retinal 1: Preparation of [1-^13^C]-, [2-^13^C]-, [1,3-^13^C_2_]- and [1,2, 3-^13^C3]-, [3-^13^C]-, [4-^13^C]-, [5-^13^C]-, [4,5-^13^C_2_]-, [6-^13^C]-, [7-^13^C]-, and [18-^13^C]- retinal 

2.3. Incorporation of ^13^C at all positions of retinal 1: Preparation of [U-^13^C]-retinal

3. Isotope Enriched Chemically Modified Retinals

3.1. Preparation of (11*Z*)-3,4-didehydroretinal, (3*R*)-(11*Z*)-3-hydroxyretinal and (4*R*)-(11*Z*)-4-hydroxyretinal

3.2. Preparation of 10-methylretinal, 10-methylthioretinal, 10-iodoretinal and 19-fluororetinal 

3.3. Preparation of 11-methylretinal and 12-methylretinal 

3.4. Preparation of 9-demethyl-9-haloretinals and 13-demethyl-13-haloretinals

4. α-Retinals *via*α-Ionone

4.1. 9-Demethyl-9-halo-α-retinals, 9-substituted α-retinals, 9-demethyl α-retinal, 19,19-ethano-α-retinal, 19,19- dimethyl α-retinal and 12- and 14-halo substituted α-retinals

5. Nor-Retinals

5.1. 16,17,18-Trinor-retinal, 16,17-dinor-retinal and 16-nor-retinal 

6. Bridged and Demethyl Retinals

6.1. DL-8,16-Methanoretinal, 8,18-methanoretinal, (*R*)-5-demethyl-8,16-methanoretinal, 1,5-didemethyl-8,16-methanoretinal, 1,1-didemethyl-8,18-methanoretinal, 1,1-didemethyl-18-didehydro-8,18-methanoretinal

6.2. 11,19-10,20-Dimethanoretinal, 10,20-methanoretinal, 13-demethyl-10,12-ethanoretinal, 13-demethyl-12,14-ethanoretinal, 13-demethyl-10,12-propanoretinal and 13-demethyl-12,14-propanoretinal

6.3. 13-Demethyl-10,14-thiaretinal and 11,14-bridged 13-demethyl retinals

6.4. 9-Demethyl retinal, 13-demethyl retinal and 9,13-didemethyl retinal

Conclusion

## Part A. Introduction

**Scheme 1:**
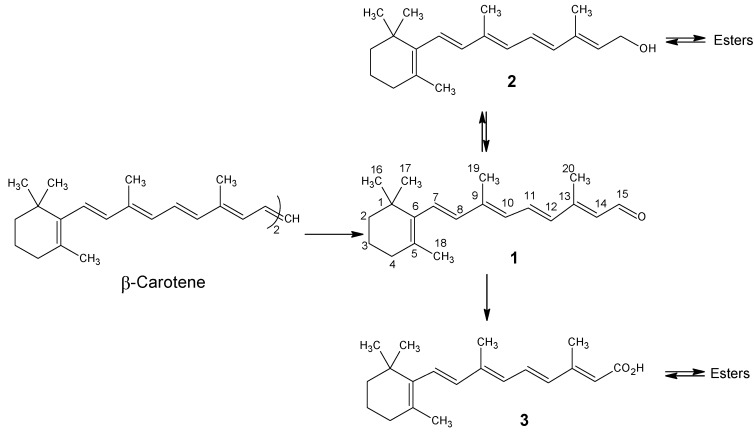
Enzymatic conversions in the human (animal body); **1**= all-*E* retinal, **2**= all-*E* retinol (vitamin A_1_), **3**= all-*E* retinoic acid. The IUPAC numbering is given in retinal **1**. Chemically all-*E* retinal **1** can be reduced by NaBH_4_ into all-*E* retinol **2**. All-*E* retinol **2** can be oxidized by MnO_2_ to all-*E* retinal **1**. All-*E* retinal **1** can be oxidized by Ag_2_O into all-*E* retinoic acid **3** and all-*E* retinoic acid **3** can be reduced by LiAlH_4_ into all-*E* retinol **2**.

In 1909 a fat soluble principal obtained from egg yolk, which proved essential for life was described [1]. Shortly afterwards, the same factor was found in butter, egg yolk extract and cod liver oil [2]. In 1919 it was observed that carotenoids have the same growth promoting activity as the factor described above, which was later called vitamin A_1_ (even later retinol) [3]. Of the thirteen natural occurring carotenoids that enter our body *via* food, four compounds have intact a β-carotene-half. They are α-carotene, β-carotene, γ-carotene and β-cryptoxanthin. These are enzymatically converted in the intestinal wall into vitamin A_1_ [4]. β-Carotene is the best source of vitamin A because it is symmetrical and the oxidative splitting of the 15,15’-double bond gives two vitamin A molecules (see Scheme 1) [5].

Interestingly, carotene biosynthesis takes place in the chloroplast of the plant cells, cyanobacteria and algae *via* the methyl erythritol phosphate pathway distinct from the mevalonic acid pathway for all other isoprenoids [6]. In 1931 the structures of β-carotene and vitamin A_1_ (retinol **2**) were published [7,8]. These structures are depicted in Scheme 1. Somewhat earlier the bioconversion of β-carotene into retinol had been described [9]. In 1944 the release of vitamin A aldehyde (retinal **1**) after the irradiation of the retina was observed [10]. The structure of the chromophore in visual pigments could be established *via* synthetic 11*Z*-retinal [11]. The visual pigments of the three phyla of animals (vertebrates, mollusks and anthropods) are membrane-bound photoreceptors that have 11*Z*-retinal as a chromophore in the active site [12]. Only in the case of amphibians and fresh water fish (11*Z*)-3,4-didehydroretinal (also known as vitamin A_2_ aldehyde) forms the chromophore. In some flies and insects the chromophore is (3R)-(11*Z*)-3-hydroxyretinal and in squid it is (4*R*)-(11*Z*)-4-hydroxyretinal [13,14].

The visual pigments belong to the important class of G-protein coupled receptors [15]. The 11*Z*-chromophore acts as the inverse agonist. Light converts it to the all-*E* structure which acts as a full agonist. In the human retina there are 120 million rods and some 6 million cones. The rods have λ_max _498 nm and they are active in scotopic vision. The three different types of cones serve photopic vision and their λ_max_ values are 425 nm, 530 nm and 560 nm, respectively. The visual pigments are located in the outer segments of the rod and cone cells. In the case of humans (and other vertebrates) at the end of the visual cycle all-*E* retinal is released from the light-activated pigment, which is reduced to all-*E* retinol. All-*E* retinol is transported to the pigment epithelium layer where it is enzymatically converted to 11*Z*-retinol, oxidized to 11*Z*-retinal and recombined with the apoprotein opsin in the outer segments to reform rhodopsin [16]. Besides being members of the important class of G-protein coupled receptors, visual pigments also belong to the retinal proteins which even occur in single cell organisms. In these organisms all-*E* retinal serves as an essential light-absorbing chromophore.

Retinoic acid **3** is an important morphogen that regulates many biological development processes in the embryo, the development of the central nervous system *etc.* Retinoic acid **3** is the agonist for the nuclear retinoic acid receptor that occurs in subforms- α, β and γ. They activate the transcription of target genes [17]. Also, a distinct type of retinoid X receptor with a similar activity has been discovered. The principal ligand for this receptor is 9*Z*-retinoic acid. These receptors (retinoic acid receptor and retinoid X receptor) form heterodimers and interact with other receptors. The status of the research in this field up to 1994 is discussed in the literature [17].

**Figure 1:**
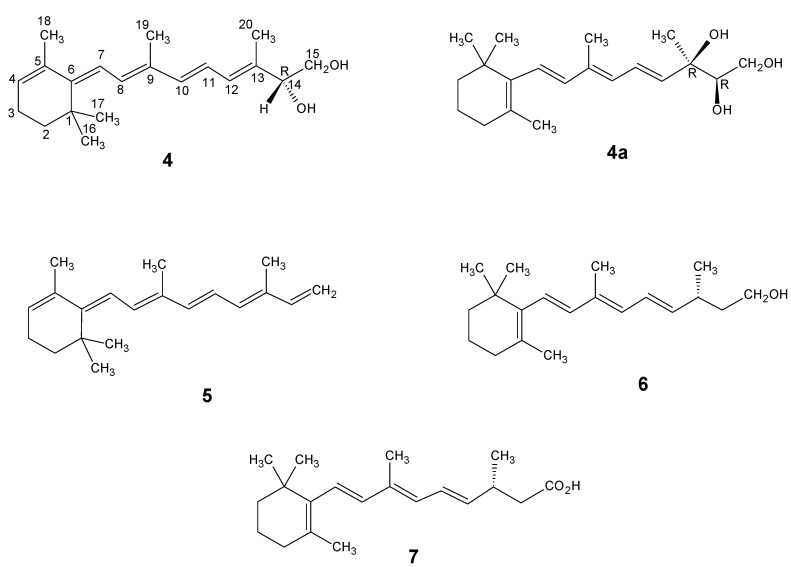
The structures of (14*R*)-14-hydroxy-4,14-*retro*-retinol **4**, (13*R*,14*R*)-13,14-dihydroxyretinol **4a**, 4,5-didehydro-15,5-*retro*-deoxyretinol or anhydroretinol **5**, (13*R*)-13,14-dihydroretinol **6** and (13*R*)-13,14-dihydroretinoic acid **7**. The IUPAC numbering is given in the structure **4**.

Since then new and important retinol metabolites in the growth control of β-lymphocytes have been reported. They are (14*R*)-14-hydroxy-4,14-*retro*-retinol **4**, all-*E* (13*R*,14*R*)-13,14-dihydroxyretinol **4a**, 4,5-didehydro-15,5-*retro*-deoxyretinol (also known as anhydroretinol) **5**, and all-*E* (13*S*,14*R*)-13,14-dihydroxyretinol (a minor diastereoisomer) [18,19,20]. Their structures are depicted in Figure 1.

Anhydroretinol **5** reversibly inhibits the action of retinol **2** and (14R)-14-hydroxy-4,14-*retro*-retinol **4**. This inhibitory function is probably due to the competition of two structurally closely related compounds for the same receptor [19]. These systems are messenger molecules that act independently from the retinoic acid receptors. Recently, the enzymatic formation of (13R)-13,14-dihydroretinol **6** has been described [21,22]. It has been observed that (13R)-13,14-dihydroretinol **6** is enzymatically converted into (13R)-13,14-dihydroretinoic acid **7**. This molecule activates the retinoic acid receptor/retinoid X receptor heterodimers but not the retinoid X receptor homodimers [23]. The study of retinol and its metabolites and their various important biological functions has been a very fruitful active research area and is steadily yielding new important findings.

Sufficient retinol or retinol equivalents have to be available *via* food to maintain the essential functions discussed before. It has been proposed that the adequate retinol amount for men and women is 700 μg and 600 μg, respectively as a daily dose [24]. Also, the retinol needs in infants, children, the elderly, pregnant and lactating women have been quantified [24]. At what intake level retinol toxicity is effective, especially in early pregnancy is not known.

When the human body doesn’t get sufficient amounts of retinol, the first observed effect is the impairment of scotopic vision leading to the night blindness. The next effect with lower levels of retinol is perturbation of the maintenance and maturation of the corneal epithelium. In many cases a so called Bitot’s spot is formed on the cornea. The molar concentration of retinol in human tears has been reported to be 0.5 × 10^-7^ [25]. The transport of retinoids in tears takes place *via* the human tear protein lipocalin [26]. Insufficient amount of retinol eventually leads to further severe health effects until the death of the organism. In 1994 it was estimated that 1.5 million children between 6–15 years of age became blind and an additional 0.5 million new cases of childhood blindness occur annually [27]. Approximately 70% of these cases are the result of severe vitamin A deficiency [28]. A later estimate is that 250 million preschool age children, particularly in developing countries are compromised by vitamin A deficiency. About 3 million children annually are likely to exhibit clinical eye signs of deficiency and between 250,000 and 500,000 may needlessly become blind [29]. Subclinical vitamin A status is difficult to access quantitatively through field surveys.

The need for sufficient vitamin A supplies started the search for commercial total synthesis by industrial firms. The first commercial supplies of vitamin A were started when Unilever decided in 1950 to use synthetic vitamin A from Hoffmann-La Roche to enrich their margarine [30]. In 1973 BASF reported that they had realized a factory that produced 600 tons of vitamin A per year *via* Wittig chemistry [31]. In 1991 it was reported that the three companies (Hoffmann-La Roche, BASF and Rhône-Poulenc) that were marketing vitamin A at that time produced about 3000 tons of vitamin A in total each year. At that moment they sold 1 kg of vitamin A for an amount of US $ 120 [32]. About 80% of the vitamin A is used in the animal feed for the intensive animal husbandry and 20% is used as human food supplement. This means that for the people in the developed world that can read these lines practically all the vitamin in the chromophore of a visual pigment is derived from industrial sources.

The main commercial vitamin A producing companies are Hoffmann-La Roche and BASF. The knowledge obtained *via* these pioneering syntheses has been essential for the further synthetic efforts in the vitamin A field. The four other commercial syntheses that are not used any more are reviewed in the literature [30,33]. Lately, the fine-chemical department of Hoffmann-La Roche including the vitamin A production has been taken over by DSM.

In order to follow the biochemical conversions in the body isotopically labeled systems are essential. This type of study was pioneered by Schoenheimer with stable isotope labeled macronutrients [34]. At that time for the micronutrient retinol this approach would not have worked because of the low sensistivity of the detection techniques for the stable isotopes. However, the radioactive isotopes ^3^H and ^14^C became available in ever higher specific activities. The preparation of ^3^H and ^14^C labeled retinoids has been reviewed [35].

In the mean time the analytical techniques have greatly improved in sensitivity such that site directed stable isotope retinoids with high isotope incorporation at at least 3 positions {(10,19,19,19-^2^H)-retinyl acetate} have become very favorable [36]. The separation properties may even change upon introduction of a number of isotopes, as in the case of deuterium enriched β-carotene; octadeutero β-carotene can be separated from natural abundance β-carotene with HPLC techniques [37].

In order to minimize isotope effects in multiisotope enriched systems the use of multi ^13^C-enriched system is very useful. The mass ratio of carbon is 13/12 and the isotopes are situated in the molecular skeleton. For these reasons ^13^C labeled systems are used recently as a reference in nutritional studies [38,39,40].

The ultimate goal in nutritional research, namely the individual establishment of the nutritional status in real time, is now in sight. The structural and functional research in visual pigments is another area where the access to a whole library of stable isotopes (^2^H, ^13^C) with a high level of site-directed incorporation is essential.

In visual pigments the chromophore (retinal) is a small part in the centre of a membrane protein, where the photochemical conversion is effected within 200 fs leading to a primary photoproduct that has a life time in the eye for about 10 ns and it is metastable below -140 °C. Resonance Raman spectroscopy gives vibrational information about the chromophore without interference of the rest of the protein due to the fact that some vibrations that are coupled to the electronic transitions may be intensified up to a million fold, compared to those not coupled to the electronic transitions. The presence of isotopes doesn’t change the molecular force field, only changes in frequencies and in coupling of vibrations occur upon introduction of heavy isotopes. The use of a set of isotopomers is essential to a full vibrational analysis and translation of this information in the structures of the starting system, the photoproduct and the various intermediates in the photocycle [41].

The other vibrational method is Fourier Transform IR difference spectroscopy, in this way the changes in vibrations between two states can be observed [42]. For example, deuterated retinal was incorporated into *p*pr (*pharaonis* phoborhodopsin), and the C-D vibrations of deuterated retinal at position 14 was examined at 2200-2300 cm^-1^ [43]. On the other hand, incorporation of ^13^C allows the use of ^13^C NMR. Rhodopsin with fully ^13^C-enriched chromophore gives the chemical shift values of each carbon atom in the chromophore. The values of the sp^2^ carbons are very useful because they supply information on the electronic charges in the chromophore [44]. Using double quantum NMR techniques of the chromophore selective for next neighbour ^13^C pairs at low temperatures, the chemical shift values of most of the sp^2^ atoms of the primary photo intermediate bathorhodopsin have been measured [45]. Torsion angles and carbon-carbon bond length in proteins can be accurately measured as well [46,47]. Specific interactions with amino acid residues in the active site next to the retinal chromophore can be detected [48].

The primary structures of all human proteins are now available with the completion of the human genome project [49]. In the post-genomic era in a very rapid process, the total genomes of a plethora of other organisms are also becoming available in addition to mutants that lead to malfunctioning or nonfunctioning proteins leading to genetic diseases. Furthermore, efficient procedures are available *via* biotechnology to obtain the proteins using these genetic codes [50,51]. The fundamental challenge now is to study the chemical processes of these proteins involving (bio)macromolecules without perturbation in the native states at the atomic level in time scales ranging from femtoseconds up to days [52].

Nature provides us with the ultimate probe *via* stable isotopes. Isotopes combine nearly identical chemical properties with different physical properties [53]. Study of a system with site-directed isotope labeling with a high incorporation level allows the determination of the whole force field *via* vibrational techniques such as FT infrared spectroscopy and (Resonance) Raman spectroscopy based on the difference in isotopic mass [41,54,55]. These techniques probe, for instance, the electron density in chemical bonds of the isotope labeled molecule. Another spectroscopic method is solid-state magic angle spinning (MAS) NMR spectroscopy, which probes the electron density at the atoms. This technique detects electronic charges at the atoms, protonation states, and configurations and conformations around bonds of the stable isotopically labeled molecule [44,56]. Comparison of the structural parameters obtained *via* these techniques for intermediate I and intermediate I + 1 in the biochemical process of the studied system provides functional information, that is, changes in protonation states, bond lengths, configuration, and conformation around bonds on the time scale involved [41,57]. When sufficient structural and functional information at the atomic level of the native form has been obtained, a whole new dimension can be attained by studying in a similar fashion systems with mutations in the protein chain and systems with rationally designed chemical changes in the cofactors [44,57]. These studies will lead to an even deeper understanding of the biochemical process. The implementation of the above-mentioned program has now utmost urgency. Without this program the now increasingly available genetic information in the post-genomic era cannot be translated into the required structural and functional information that will lead to the expected quantum jump in the understanding of the various processes in human (animal) health and diseases and the expected rational approach to treat these diseases. The access to a full number of possible site-directed stable isotopically enriched building blocks (amino acids and cofactors) up to the uniformly labeled systems is a “condition sine qua non” for the proposed structural and functional investigations. All uniformly isotopically labeled amino acids and several cofactors are available *via* photosynthetic organisms that are grown in media containing ^13^CO_2_ and ^15^NH_3_ [58]. To start the above-mentioned program, access to building blocks with isotope labels at each defined atomic position and combination of positions up to the uniformly labeled form is required. The only way to obtain access to the whole cassette of desired isotopomers is a modular synthetic approach such that one synthetic scheme can give in a rational way the required building block as a cassette of all isotopomers. This approach may seem Herculean; however, only 20 different amino acids and a limited number of cofactors are required. The synthesis of these cassettes has to be based on a limited number of commercially available highly enriched stable isotopically labeled starting materials.

The selection of [U-^13^C_20_]-labeled all-*E* retinal and all its isotopomers in the new modular approach is based on the fact that retinal and retinoids play a very important role in many life processes [59,60,61]. In addition, structural and functional studies with isotope sensitive techniques have already been initiated in the field of rhodopsin [44,56,62,63].

Rhodopsin serves as the paradigm for the superfamily of seven transmembrane helix G-protein coupled receptors (GPCRs) [64]. The GPCRs mediate a broad array of physiologically and pharmacologically important signal transduction processes. GPCRs trigger a wide variety of physiological processes that involve signaling by neurotransmitters, hormones, and neuropeptides [65]. Consequently, GPCRs are the major pharmaceutical targets for pharmacological intervention in human (and veterinary) pathology. In rhodopsin the photoreactive ligand is 11*Z-*retinal that is covalently bound in the interior of the protein *via* a protonated Schiff base linkage with lysine residue 296 to form the retinylidene chromophore [66].

The full vibrational analysis of the chromophore of rhodopsin and its photoproduct bathorhodopsin has been reported *via* about 70 isotopomers [55,67]. Furthermore, the chemical shift values of the sp^2^ carbons in the tail end of the chromophore have been reported [44,62]. The distances between C_10_-C_20_ and C_11_-C_20_ could be determined with very high precision (0.1 Å) *via* the 1-D rotational resonance solid-state ^13^C NMR technique [56]. From these distance measurements the precise chromophore structure could be derived. The molecular torsional angle around a bond in the chromophore labeled with two ^13^C isotopes could be directly established *via* this method [68]. Furthermore, the ultrahigh-field solid-state MAS NMR study on the [8,9,10,11,12,13,14,15,19,20-^13^C10]-11-*Z*-retinylidene chromophore in its natural lipid membrane environment has been published [44]. This study showed that the use of multispin labeling in combination with 2-D solid-state MAS NMR correlation spectroscopy improves the relative accuracy of the shift measurements in solids. This allows the electronic structure of the retinylidene chromophore to be analyzed at high levels of understanding: (1) by specifying the interactions between the ^13^C-labeled ligand and the G-protein coupled receptor target and (2) by making an assessment of the various factors contributing to the charge distribution in the chromophore. Nowadays, information of much higher quality about 20 carbon atoms can be obtained in one short experimental session that thus far has taken a decade to collect [69]. Nevertheless, these results are obtained *via* studies of the almost unlimited supply of bovine rhodopsin from cattle eyes. However, cone pigments of man and other animals and the various opsins with site-directed mutations have to be obtained *via* biotechnological expression systems, which implies that now the availability is the limiting factor [51,70]. Recently, the solid-state ^15^N MAS NMR study of rod visual pigment rhodopsin in which 99% ^15^N enriched [α,ε-^15^N_2_]-L-lysine is incorporated by using the baculovirus/Sf9 cell expression system has been published [63].

In this paper the synthetic routes adopted by DSM and BASF for the production of vitamin A will be discussed in part B. Then, the synthesis of deuterated retinol with CD or CD_3_ groups on the conjugated chain and the strategies to obtain retinals enriched with ^13^C on a limited number of positions (for the accessibility of many mono- and di-^13^C-enriched retinals) will be discussed in part C. Finally, the schemes that allow preparation of any ^13^C isotopomer will be reviewed. This will be complemented by chemically modified retinals that are now accessible in any isotopically labeled form. The paper closes with the discussion of chemically modified retinals where access to any isotopomer has not yet been attained. The synthetic retinoids by other groups have been extensively reviewed in literature [68,69,70,71]. Retinoids can occur in 16 different geometric isomers. The access to the whole set of geometric isomer has been described [71,72,73,74].

## Part B. Technical Syntheses

## 1. Hoffmann-La Roche, DSM

**Scheme 2:**
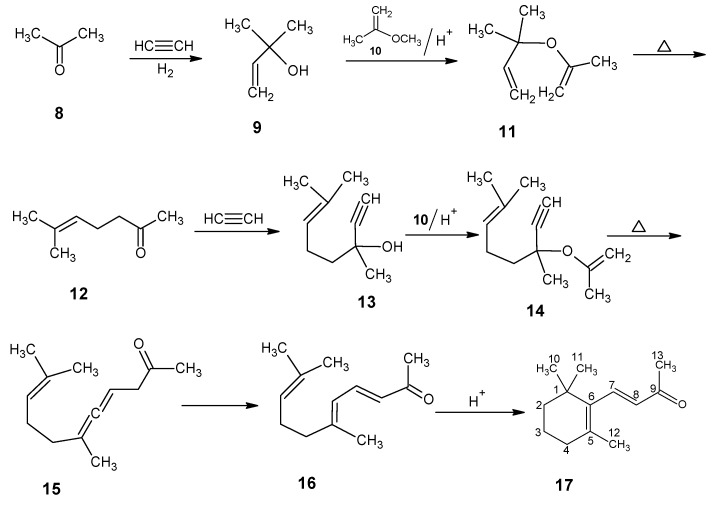
The technical conversion of acetone **8** and acetylene into β-ionone **17**.

In Scheme 2 the synthetic route is depicted to show the Hoffmann-La Roche, DSM technical synthesis of vitamin A from the simple starting materials that lead to β-ionone **17**, an important intermediate in the commercial production of vitamin A [33,75,76]. For a technical process to be commercially viable a synthetic scheme has to be developed that starts with simple available starting materials from petrochemical sources.

Acetone **8** is coupled with the acetylide anion (prepared by the reaction of acetylene and Grignard reagent) to give 2-methylbut-3-yn-2-ol which is reduced by Lindlar catalyst (selective hydrogenation) to 2-methylbut-3-en-2-ol **9**. This product is coupled with 2-methoxyprop-1-ene **10** under acid catalyzed condition to give propenyl ether **11** [77]. An oxy-Cope rearrangement of the propenyl ether **11** gave 6-methylhep-5-en-2-one **12**. Further coupling of the product **12** with the acetylide anion gave 3,7-dimethyloct-6-en-1-yn-3-ol **13**. Treatment of the product **13** with 2-methoxyprop-1-ene **10** upon acid catalyzed condition afforded 3,7-dimethyl-3-(prop-1-en-2-yloxy)oct-6-en-1-yne **14**, which underwent a Cope rearrangement to give the allenic ketone **15**. Compound **15** is isomerized to pseudoionone **16** [78]. Pseudoionone **16** is cyclized into β-ionone **17** in the presence of sulphuric acid. In this reaction protonation takes place at carbon 2 to give tertiary carbenium ion at carbon 1. Carbon-carbon bond formation takes place to give a six membered ring by nucleophilic attack of carbon 6 at the carbon 1 (the tertiary carbenium ion) and further work-up afforded β-ionone **17**.

**Scheme 3:**
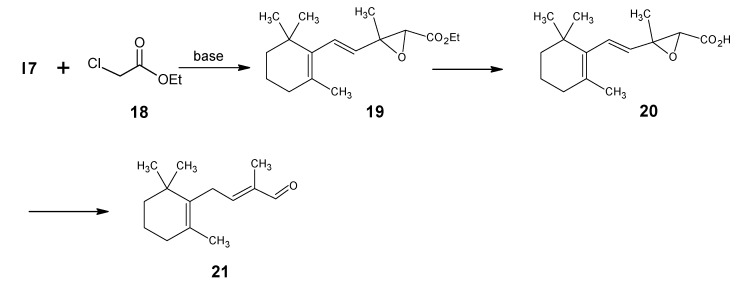
The technical conversion of β-ionone **17** into β-C_14_ aldehyde **21**.

The reactions depicted in Scheme 3 are used for the one carbon chain extension of β-ionone **17** into the β-C_14_ aldehyde {2-methyl-4-(2’,6’,6’-trimethylcyclohex-1’-en-1’-yl)but-2-en-1-al} **21**. A Darzens condensation of **17** with ethyl chloroacetate **18** gave the glycidic ester **19**. A base induced saponification of the product **19** followed by an acid catalyzed epoxy ring opening of the product **20** and expulsion of CO_2_ gave the β-C_14_ aldehyde **21**.

**Scheme 4:**
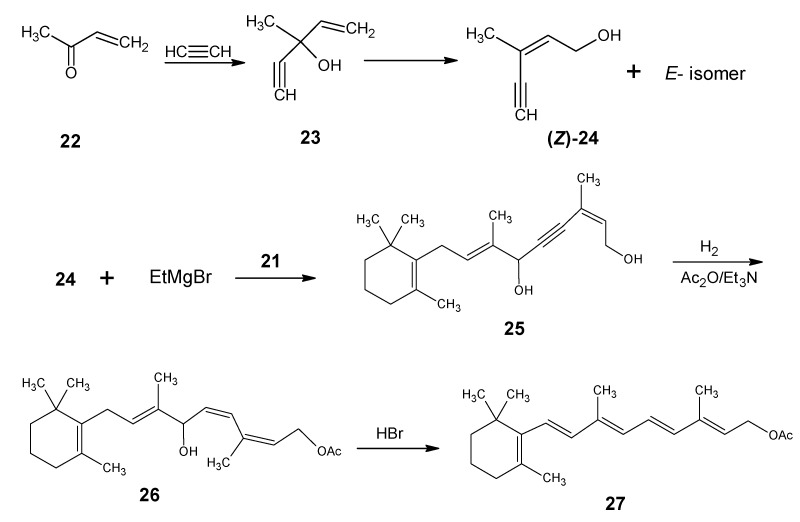
The preparation of (2*Z*/2*E*)-3-methylpent-2-en-4-yn-1-ol **24**, coupling of **24** (*Z*-isomer) with β-C_14_ aldehyde **21** to give the product **25** and final conversion of the product **25** into all-*E* retinyl acetate **27**.

The synthetic route depicted in Scheme 4 is used to prepare (2*Z*)-3-methylpent-2-en-4-yn-1-ol **24**. Methyl vinyl ketone **22** is treated with the acetylide anion to form 3-methylpent-4-en-1-yn-3-ol **23**. Acid catalyzed rearrangement gave a mixture of (*E*/*Z*)-3-methylpent-2-en-4-yn-1-ol **24**. *Via* distillation (2*Z*)-3-methylpent-2-en-4-yn-1-ol **24** is isolated in pure form. Treatment of (*Z*)-enynol **24** with two equivalents of Grignard reagent gave the dianion of **24** which is coupled with β-C_14_ aldehyde **21** to give the diol **25** with full vitamin A skeleton. Subsequent Lindlar reduction gave the *cis*-diol product which is selectively acetylated on the primary hydroxyl function to give the acetate derivative **26**.

Acid catalyzed elimination of the hydroxyl group from carbon 10 of the product **26** led to a heptatrienyl cation encompassing the sp^2^ atoms from 8 to 14. In this fully conjugated system the bond order of each carbon-carbon bond is 1.5 leading to dynamic *E*/*Z* isomerization resulting in the most stable fully extended system. Subsequent loss of a proton from carbon 7 gave retinyl acetate **27** in the all*-E* form in high yield. For information about carbenium ions in the retinoid field see literature [79]. Stronger acid will lead to the protonation at carbon 14 in vitamin A acetate (retinyl acetate) to form a conjugate carbenium ion that will lose a proton from carbon 4 in the ring to form *retro*-vitamin A acetate (this is the compound **4** in Figure 1 without the hydroxyl group at carbon 14).

Similarly, the absence of acetyl protection in the product **26** will give anhydroretinol **5** *via* elimination of two molecules of water. It is remarkable that the mild acid treatment of the product **26** on large scale leads to the required retinyl acetate **27** in high yield without appreciable *retro*-vitamin A formation. In this technical process Grignard reagents are used on a large scale, for example batches containing 500 kg of magnesium are used; as a result a great amount of magnesium salt is obtained as a waste. Many steps need only small amount of Pd and acid as a catalyst to minimize the formation of waste product.

## 2. BASF

**Scheme 5:**
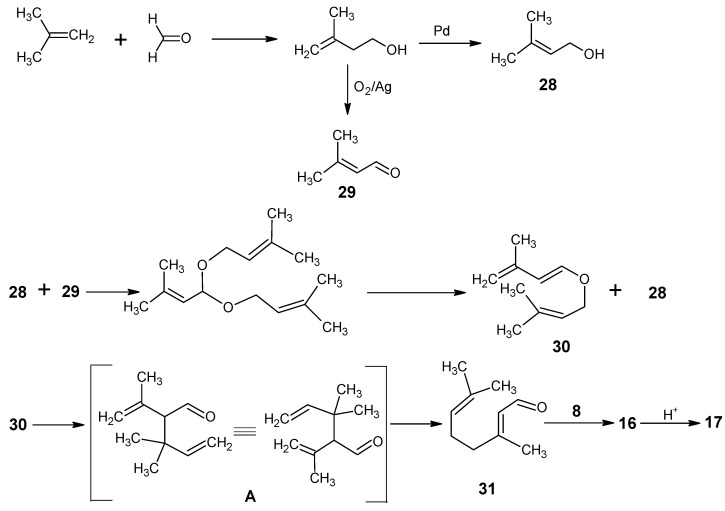
The conversion of isobutene and formaldehyde into β-ionone **17** *via* citral **31**.

**Scheme 6:**
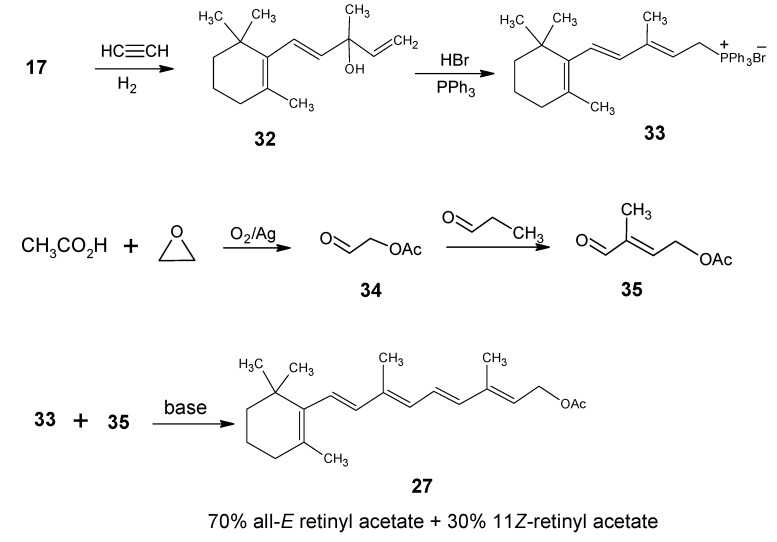
Preparation of [2-(β-ionylidene)ethyl]triphenylphosphonium bromide **33** and all-*E* 4-acetoxy-2-methyl-but-2-en-1-al **35** and the final HWE coupling of **33** and **35** into **27** (70% all-*E* retinyl acetate and 30% 11*Z*-retinyl acetate).

The technical synthesis of vitamin A developed by BASF starts with the reactions depicted in Scheme 5 and Scheme 6 [80,81,82]. Acid catalyzed reaction of isobutene and formaldehyde gave 3-methylbut-3-en-1-ol. Pd catalyzed isomerization converted it into the allylic alcohol **28** and Ag catalyzed oxidation gave 3-methylbut-2-en-1-al **29**. Products **28** and **29** under azeotropic condition in the presence of nitric acid formed an acetal which eliminated one molecule of **28** at higher temperatures. The intermediate enol ether underwent oxy-Cope rearrangement to give the citral **31**. Pseudoionone **16** is obtained by an aldol condensation of citral **31** with acetone **8**. The product **16** is cyclized in the presence of an acid to give the β-ionone **17**. The acid catalyzed conversion of **16** to **17** has been discussed in Scheme 2.

In Scheme 6 the conversion of β-ionone **17** into vitamin A acetate **27** is depicted. Reaction of β-ionone **17** with the acetylide anion and subsequent Lindlar reduction gave the vinyl β-ionol **32**. Treatment of **32** with HBr in the presence of triphenylphosphine gave all-*E* conjugate carbenium ion (as has been discussed before). The presence of the soft base triphenylphosphine attacks preferentially at the position 11 to form the all*-E* [2-(β-ionylidene)ethyl]triphenylphosphonium bromide **33** [83]. Triphenylphosphine is prepared in an industrial scale by reacting PCl_3_ (obtained by the reaction of phosphorous and chlorine) and phenylsodium (obtained by the reaction of phenyl chloride and sodium).

4-Acetoxy 2-methylbut-2-en-1-al **35** is the required C_5_ building block for the preparation of vitamin A acetate **27**. Oxirane is treated with acetic acid in the presence of catalytic Ag and air leading to 2-acetoxy ethanal **34**. Product **34** underwent an aldol condensation with propanal to give all-*E* 4-acetoxy-2-methylbut-2-en-1-al **35**. Treatment of **33** with methanolate gave an ylide which is coupled with all-*E* **35** to afford retinyl acetate **27 (**70% all*-E* and 30% 11*Z*). This means that during the coupling the geometric integrity of the bonds is maintained. Only the new carbon-carbon double bond (C11-C12) is formed both in the predominant *E* and in the minor 11*Z*-isomer. The latter can easily be converted into the all*-E* form. Later in the literature the use of 1,2-epoxybutane as a pseudo base in Wittig reactions has been described [84]. In this case the bromine anion attacks the epoxy ring to form an alcoholate transiently which removes the proton from the phosphonium salt. In this method the Wittig condensation is carried out under very mild conditions.

In this technical process also catalytic reactions are used to minimize the side products. A significant contribution to the cost is the preparation of triphenylphosphine at an industrial scale. The resulting triphenylphosphine oxide has a very strong P-O bond which makes reconversion into triphenylphosphine expensive and difficult. Initially, the triphenylphosphine oxide was reconverted into triphenylphosphine and reused. Nowadays the residual triphenylphosphine oxide is pyrolysed.

## Part C. Site-Directed Highly Stable Isotope Enriched Retinals

## 1. Deuterium Labeled Retinals

Highly enriched (99%) starting materials available for deuterium incorporation are D_2_O, LiAlD_4_, NaBD_4_, CD_3_I, CD_3_CN, (CD_3_)_2_CO. Especially with deuterium incorporation the reactions used in the synthetic procedures should not lead to deuterium loss or scrambling. Also, after the primary deuterium incorporation the number of synthetic steps should be minimal with possible conversions to get the target molecule in a reasonable yield.

### 1.1. Incorporation of deuterium at positions 18 and 19 of retinal 1: Preparation of 11Z- [18-D_3_]-retinal and 11Z-[19-D_3_]-retinal via [12-D_3_]-β-ionone 17a and [13-D_3_]-β-ionone 17b, respectively

**Scheme 7:**
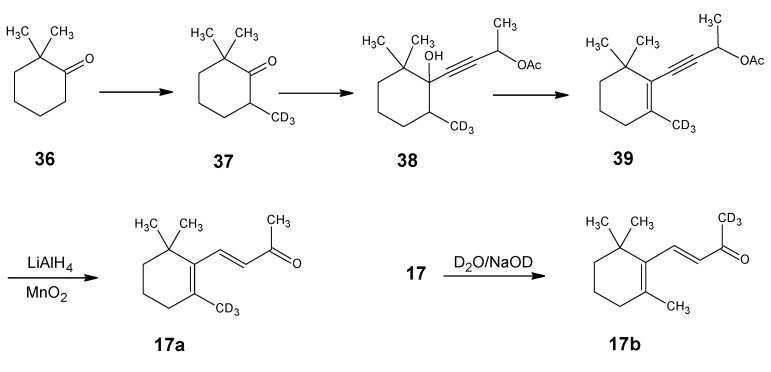
Conversion of 2,2-dimethylcyclohehexanone **36** into [12-D_3_]-β-ionone **17a**. Base catalyzed conversion of β-ionone **17** into [13-D_3_]-β-ionone **17b**. IUPAC numbering of β-ionone **17** is given in Scheme 2.

The reactions shown in the synthetic route in Scheme 7 are used for the preparation of [12-D_3_]-β-ionone **17a** and [13-D_3_]-β-ionone **17b** [85]. The starting material 2,2-dimethylcyclohexanone **36** is treated with methanolate base to afford the anion of **36** which upon reaction with CD_3_I a mixture of mono and bistrideuteromethylated products is obtained together with residual **36**. After purification the product **37** is treated with the dianion of 1-butyn-3-ol (prepared by the reaction of 1-butyn-3-ol with 2 equivalents of EtMgBr) to obtain the product **38** with the full carbon skeleton of β-ionone.

**Scheme 8:**
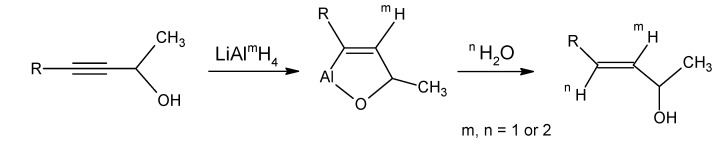
Specific protonation or deuteration in the conversion of a propargylic alcohol into an allylic alcohol, (m= 1, LiAlH_4_; m= 2, LiAlD_4_; n= 1, H_2_O; n= 2, D_2_O).

The secondary hydroxyl group is acetylated and the tertiary hydroxyl group is eliminated by the treatment with POCl_3_/pyridine to give the enyne **39**. Reduction of triple bond of the product **39** with LiAlH_4_ and H_2_O according to Scheme 8 afforded [12-D_3_]-β-ionol which is converted into the corresponding [12-D_3_]-β-ionone **17a** upon MnO_2_ oxidation. For the conversion of [12-D_3_]-β-ionone **17a** into [18-D_3_]-retinyl acetate the reactions depicted in Scheme 6 are carried out. After saponification of [18-D_3_]-retinyl acetate (a mixture of 70% all-*E* and 30% 11*Z*) the retinols are obtained which are converted into retinals *via* MnO_2_ oxidation. With preparative HPLC techniques the required 11*Z*- [18-D_3_]-retinal is separated. The all-*E* retinal in acetonitrile is treated with light (tungsten). After work-up an additional amount of 11*Z*- [18-D_3_] retinal is isolated in pure form.

The protons of the methyl group attached to carbonyl group in β-ionone **17** are acidic, twice base catalyzed hydrogen-deuterium exchange in D_2_O/pyridine afforded more than 95% trideuterium incorporation at the methyl carbon to give [13-D_3_]-β-ionone **17b**. Using **17b** as a starting material for the reactions in Scheme 6 11*Z*-[19-D_3_]-retinal with deuterium incorporation of > 99% is obtained.

### 1.2. Incorporation of deuterium at positions 10, 11, 12, 14 and 20 of retinal 1: Preparation of all-E [10-D]-retinal, all-E [11-D]-retinal, all-E [12-D]-retinal, all-E [11,12-D_2_]-retinal, all-E [10,11-D_2_]-retinal, all-E [14,20,20,20-D_4_]-retinal

It has been discussed in Scheme 6 that β-ionone **17** upon treatment with the acetylide anion afforded acetylenic alcohol. This alcohol is treated with LiAlD_4_ and subsequent work-up gave [10-D]-vinyl ionol which is converted into [10-D]-retinal following the synthetic route presented in Scheme 6 [86]. This product showed deuterium incorporation of about 82%.

**Scheme 9:**
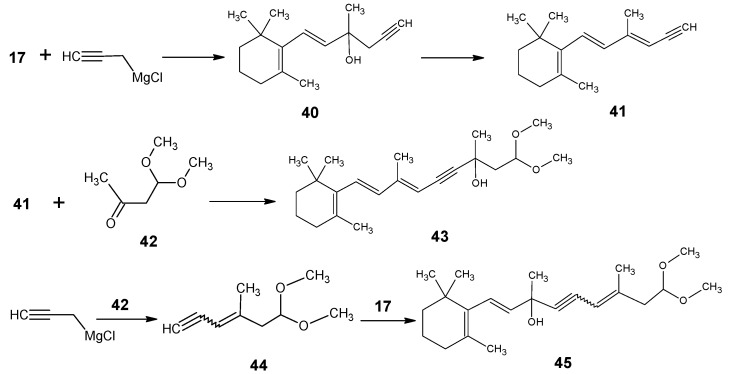
The conversion of β-ionone **17** into propargylic alcohols **43** and **45** with full retinal carbon skeleton.

For the preparation of [11-D], [12-D] and [11,12-D_2_]-retinals the propargylic alcohol **43** (triple bond between carbon 11 and carbon 12) with the full vitamin A skeleton has to be prepared *via* Scheme 9 [87]. β-Ionone **17** is treated with propargyl magnesium halide to give propargylic alcohol **40**. Dehydration of **40** gave the trienyne **41** in two isomeric forms. The anion of **41** is coupled with 4,4-dimethoxylbut-2-one **42** to give the required propargylic alcohol **43**.

The LiAlD_4_ treatment of product **43** as in Scheme 8 afforded deuterium incorporation at positions 11 and 12. Further work-up in acidic medium eliminated the alcohol function and deprotected the acetal function to give all-*E* [11-D]-retinal, all-*E* [12-D]-retinal and all-*E* [11,12-D_2_]-retinal with site-directed high deuterium incorporation. The final acid catalyzed removal of hydroxyl function and deprotection are very critical steps. However, these reactions should be performed quickly before isotopic loss and scrambling take place.

For the preparation of [10-D]-retinal, [11-D]-retinal, [10,11-D_2_]-retinal, propargylic alcohol **45** (triple bond between carbon 10 and carbon 11) are prepared. First, 4,4-dimethoxylbut-2-one **42** is reacted with propargyl magnesium halide, then water is eliminated to give the enyne **44**. This building block is converted into the anion and coupled with β-ionone **17** to give the propargylic alcohol **45** with the full retinal carbon skeleton. The deuteration/protonation and deprotection procedure as discussed above gave the required deuterated retinals. For the preparation of [14,20,20,20-D_4_]-retinal, first 4,4-dimethoxybut-2-one **42** is pentadeuterated *via* a suspension of **42** in D_2_O in the presence of a catalytic amount of K_2_CO_3_. Following the reactions mentioned in Scheme 9 and final work-up the required deuterated retinal is obtained with ca. 80% tetradeuterium incorporation.

### 1.3. Incorporation of deuterium at positions 14,15, and 20 of retinal 1: Preparation of all-E [14-D]-retinal, all-E [15-D]-retinal, all-E [14,15-D_2_]-retinal and all-E [20,20,20-D_3_]-retinal.

**Scheme 10:**
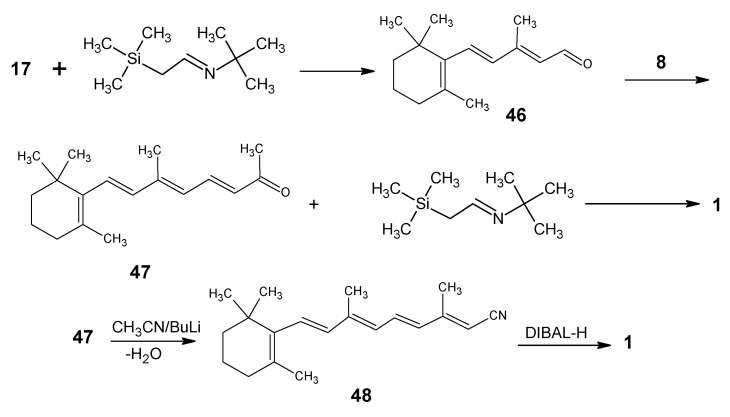
Conversion of β-ionone **17** into retinal **1**.

For the incorporation of deuterium at positions 14, 15 and 20 the reactions depicted in Scheme 10 are used. Peterson olefination of β-ionone **17** with silylcarbanion of [*N-*(2’-t-butylsilyl)-ethylidene]-2-methylpropane-2-amine gave β-ionylidene acetaldehyde **46** [88]. Base catalyzed aldol condensation with acetone **8** gave C_18_ ketone **47** which is converted into retinal **1** *via* a second Peterson Olefination reaction. Another approach is to treat **47** (C_18_ ketone) with the anion of acetonitrile (prepared by treating acetonitrile with BuLi at low temperature) and subsequent elimination of a molecule of water afforded retinonitrile **48** which upon DIBAL-H reduction afforded retinal **1**.

Use of CD_3_CN in the reaction in Scheme 10 afforded [14-D]-retinal. The conversion of [14-D]-retinal into [14-D]-methyl retinoate is achieved *via* successive treatment with MnO_2_, KCN, CH_3_OH, CH_3_CO_2_H. The methyl ester upon reduction with LiAlD_4_ yielded [14,15,15-D_3_]-retinol. Subsequent MnO_2_ oxidation gave [14,15-D_2_]-retinal. Similarly, LiAlD_4_ reduction of methyl retinoate afforded [15,15-D_2_]-retinol. Oxidation of the later with MnO_2_ gave [15-D]-retinal. To obtain [20,20,20-D_3_]-retinal hexadeuterated acetone in the aldol condensation of **46** should be used. An alternative method of deuterium incorporation at carbon 18 (methyl ketone) in product **47** is by mixing the product in D_2_O in the presence of a catalytic amount of NaOD (similar to reactions shown in Scheme 7).

## 2. ^13^C-Labeled Retinals

^13^C-Enriched retinals are prepared *via* the use of ^13^CH_3_CN, CH_3_^13^CN, and ^13^CH_3_^13^CN. Acetonitrile is commercially available in the three possible highly enriched ^13^C isotopomers.

### 2.1. Incorporation of ^13^C at positions 14 and 15 of retinal 1: Preparation of [14-^13^C]-retinal, [15-^13^C]-retinal and [14,15-^13^C_2_]-retinal

Following the synthetic route mentioned in Scheme 10 ^13^C-labeled retinonitrile is obtained by the use of ^13^C-labeled acetonitrile. DIBAL-H reduction of the corresponding nitrile function in ^13^C-labeled retinonitriles gave [14-^13^C]-retinal, [15-^13^C]-retinal and [14,15-^13^C_2_]-retinal [88]. Also, the ^13^CD_3_-enriched acetonitrile is commercially available. That means all ^2^H and ^13^C isotopomers at positions 14 and 15 of retinal **1** are accessible *via* the reactions discussed above.

**Scheme 11:**
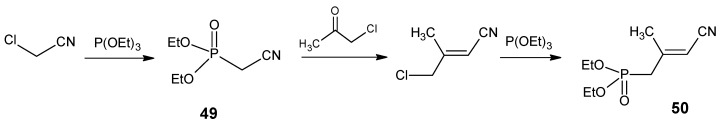
Preparation of Horner-Wadsworth-Emmons (HWE) reagents: diethyl phosphonoacetonitrile **49** and 4-(diethylphosphono)-3-methylbut-2-enenitrile **50**.

In Scheme 11 it is shown that an Arbuzov reaction of triethylphosphite and chloroacetonitrile gave diethyl phosphonoacetonitrile **49** and subsequent treatment of the product with one equivalent of lithium diisopropylamide (LDA) in THF afforded the anion of **49** *in situ*. An alternative way to obtain the anion of **49** in ^13^C isotopomeric form is by reacting ^13^C-enriched acetonitrile with two equivalents of LDA and then adding one equivalent of diethyl chlorophosphate. 4-Chloro-3-methylbut-2-enenitrile is obtained by the HWE reaction of **49** with 1-chloroacetone and further Arbuzov reaction with triethylphosphite gave a mixture of (*Z*/*E*)-4-(diethylphosphono)-3-methylbut-2-enenitrile **50**.

**Scheme 12:**
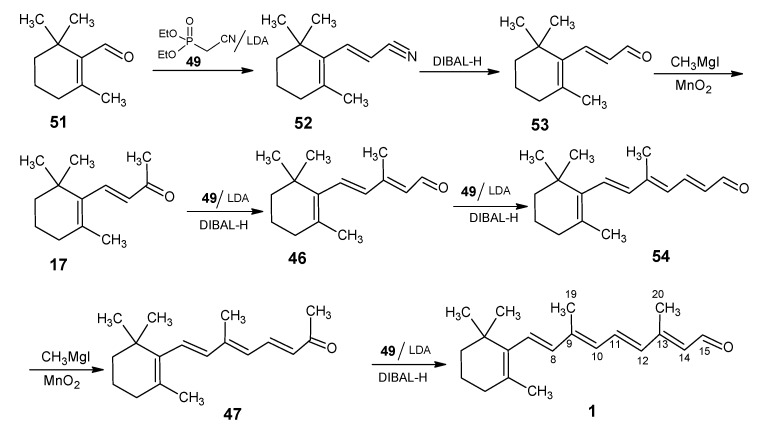
Preparation of retinals with ^13^C enrichment at positions 8,9,10,11,12,13,14,15, 19 and 20.

Retinals with ^13^C enrichment at positions 8, 9, 10, 11, 12, 13, 14, 15, 19 and 20 are accessible *via* the synthetic route depicted in Scheme 12 [89,90]. β-Cyclocitral **51** is treated with the anion of **49** to give the conjugated nitrile **52** which upon DIBAL-H reduction afforded the conjugated aldehyde **53**. The aldehyde **53** is treated with CH_3_MgI to give β-ionol which upon MnO_2_ oxidation gave β-ionone **17**. The Grignard reagent methylmagnesium iodide can also be obtained in 99% ^13^C-enriched form by treating commercially available ^13^CH_3_I with magnesium. Any ^13^C isotopomer of **17** is accessible *via* Scheme 12 by using the right sequence of isotope enriched reagents and the reagents in natural isotope abundance form.

### 2.2. Incorporation of ^13^C in the six membered ring of retinal 1: Preparation of [1-^13^C]-, [2-^13^C]-, [1,3-^13^C_2_]- and [1,2, 3-^13^C3]-, [3-^13^C]-, [4-^13^C]-, [5-^13^C]&gt;-, [4,5-^13^C_2_]-, [6-^13^C]-, [7-^13^C]-, and [18-^13^C]- retinal

**Scheme 13:**
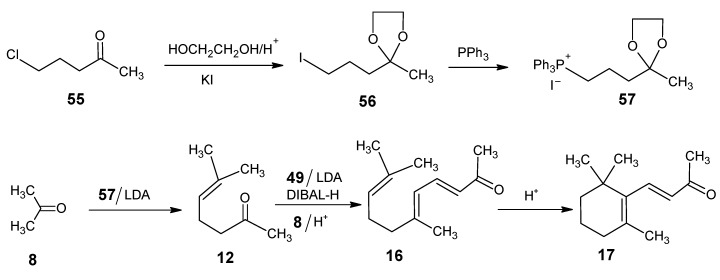
Conversion of 5-chloropenta-2-one **55** into β-ionone **17**.

In Scheme 13 the synthetic route is depicted to show that commercial 5-chloropent-2-one **55** is used as a starting material to obtain final [1-^13^C]-retinal and [16,17-^13^C_2_]-retinal. The product **55** is converted into the acetal with ethylene glycol and subsequent treatment with KI in acetone gave the iodoacetal **56**. Treatment with triphenylphosphine yielded the phosphonium iodide **57** which upon treatment with BuLi gave the ylide that coupled with acetone **8** to give the acetal of **12**. Deprotection of acetal afforded 6-methylhep-5-en-2-one **12**. After conversion of product **12** to pseudoionone **16** followed by the acid treatment afforded β-ionone **17** [91].

Acetone is available in [1-^13^C]-, [2-^13^C]-, [1,3-^13^C_2_]- and [1,2, 3-^13^C3]-isotopomeric form. In the case of the [1-^13^C]-acetone a 50/50 mixture of the [16-^13^C] and [17-^13^C] retinal is obtained. With [2-^13^C]-, [1,3-^13^C_2_]- and [1,2,3-^13^C3]-acetone well defined isotopically enriched retinals are obtained.

For the chain extension of β-ionone **17** we have used the building block **50** which is prepared *via* the synthetic route depicted in Scheme 11. In order to effect a more efficient conversion of β-ionone **17** into isotopically labeled retinals the ^13^C-enriched phosphonates **49** and **50** described in Scheme 11 are used. (*E*/*Z*)-β-Ionylidene acetonitrile is obtained by the HWE reaction of the anion of **49** with β-ionone **17** which upon DIBAL-H reduction gave the (*E*/*Z*)-β-ionylidene acetaldehyde **46** (Scheme 12). Product **46** is treated with the anion of **50** to form a mixture of geometric isomers of retinonitrile **48** which is further reduced by DIBAL-H to obtain a mixture of geometric isomers of retinal **1** (Scheme 10). The use of building blocks **49** and **50** together with subsequent nitrile to aldehyde reduction made these schemes much more efficient.

**Scheme 14:**
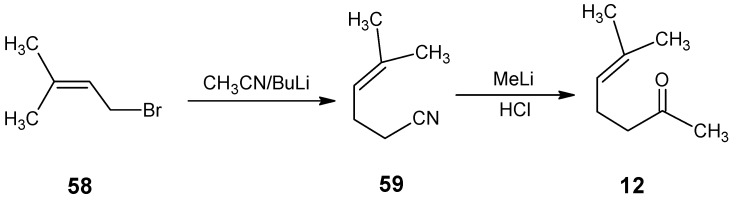
Preparation of 6-methylhept-5-ene-2-one **12**.

For the incorporation of ^13^C at positions 4 and 5 of retinal **1** the reactions in Scheme 14 are worked out. Commercially available 1-bromo-3-methylbut-2-ene **58** is treated with an equivalent of the anion of acetonitrile (prepared by 1 eq. BuLi in THF) to give 5-methylhex-4-enenitrile **59**. Treatment of **59** with methyllithium afforded 6-methylhept-5-ene-2-one **12** which is converted into β-ionone **17** *via* synthetic routes depicted in Scheme 13. By using ^13^C-enriched acetonitrile [4-^13^C]-, [5-^13^C]-, and [4,5-^13^C_2_]-retinals are obtained.

**Scheme 15:**
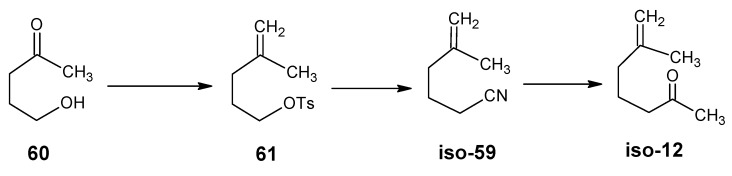
Conversion of commercially available 4-oxopentan-1-ol **60** into **iso-12**.

The reactions in Scheme 15 are used for a less expensive alternative for the preparation of [18-^13^C]-retinal. Commercially available 4-oxopentan-1-ol **60** is first acetylated, then treated with the anion of triphenylmethyl phosphonium bromide and subsequently saponified. The resulting alcohol is converted into the tosylate **61**. Treatment with KCN gave the isomer of nitrile **59** (**iso-59**) and methylmagnesium iodide converted this product into **iso-12**. This is converted into isocitral (**iso-31**) *via* the reaction with diethyl phosphonoacetonitrile **49** and subsequent DIBAL-H reduction followed by condensation with acetone **8** afforded iso-pseudoionone. Presence of an acid converted it into β-ionone **17**. Using K^13^CN, ^13^CH_3_MgI and ^13^C-labeled acetonitrile gave at the end [5-^13^C]-, [6-^13^C]-, [7-^13^C]-, and [18-^13^C]-retinals.

**Scheme 16:**
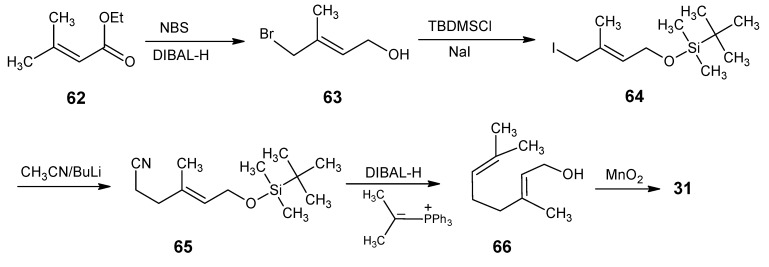
Conversion of ethyl 3-methylbut-2-enoate **62** into citrol **66**.

For ^13^C-incorporation at positions 2 and 3 of retinal the reactions of the synthetic route in Scheme 16 are followed. Commercially available ethyl 3-methylbut-2-enoate **62** *via* NBS bromination and subsequent DIBAL-H reduction is converted into the unsaturated bromoalcohol **63** [92]. Protection of the alcohol function with tert-butylmethylsilyl chloride and treatment with NaI in acetone yielded the iodide **64** which is treated with the anion of acetonitrile to give the nitrile **65**. DIBAL-H reduction of **65** and subsequent treatment with isopropylidene(triphenylphosphorane) and deprotection gave citrol **66**. Treatment of **66** with MnO_2_ afforded citral **31**. Citral **31** is converted into β-ionone **17** (Scheme 5) and finally into retinal **1** *via* the reactions mentioned in Scheme 12. Use of ^13^C-labeled acetonitrile in the reaction with product **64** afforded [2-^13^C]- and [3-^13^C]-retinals.

The Scheme 10, Scheme 11, Scheme 12, Scheme 13, Scheme 14, Scheme 15 and Scheme 16 have given possibility of a large number of site-directed ^13^C-enriched retinals. In principle all ^13^C isotopomers of retinals are now accessible. However, due to chirality of carbon 1 ^13^C-enrichment only at either position 16 or position 17 leads to inseparable mixtures of enantiomers in the final retinal **1**.

### 2.3. Incorporation of ^13^C at all positions of retinal 1: Preparation of [U-^13^C]-retinal

**Scheme 17:**
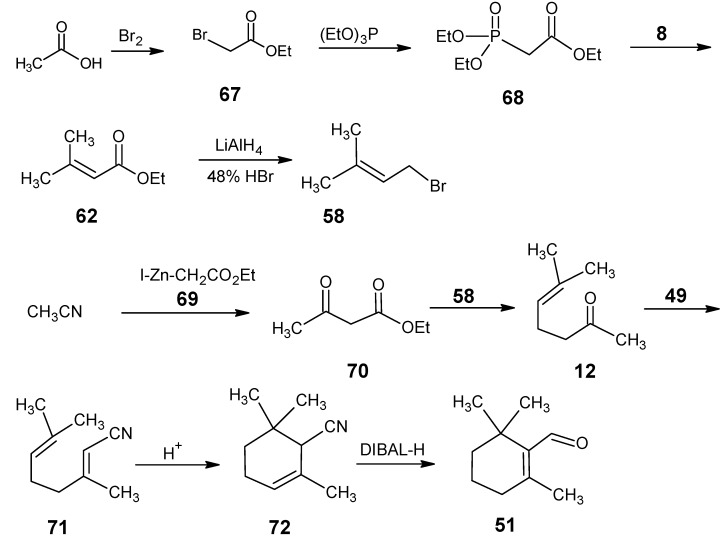
The synthesis of β-cyclocitral **51** in any site-directed ^13^C-enriched form.

In order to obtain highly ^13^C-enriched up to the [U-^13^C]-retinal a more convergent reaction Scheme with highly ^13^C-enriched building blocks is developed [52]. In Scheme 17 the reactions are depicted that lead to any site-directed ^13^C-enriched β-cyclocitral **51**. Acetic acid is converted *via* Hell-Volhardt-Zelinsky bromination and subsequent esterification into ethyl bromoacetate **67**. An Arbuzov reaction of **67** with the triethylphosphite gave ethyl diethylphosphonoacetate **68**. The HWE coupling of the product **68** with acetone **8** gave ethyl 3-methylbut-2-enoate **62**. Reduction of **62** with LiAlH_4_ and subsequent bromination of the allylic alcohol afforded 1-bromo-3-methylbut-2-ene **58**. Ethyl 3-oxobutanoate **70** is commercially available in all ^13^C_4_ form.

All other isotopomers are prepared *via* the Blaise reaction of acetonitrile with the ethyl zinc-iodoacetate **69**. Ethyl iodoacetate is obtained by the reaction of product **67** with KI in acetone which is further treated with zinc to obtain product **69** *in situ*. Base induced coupling of **58** and ester **70** afforded the product which after saponification and acid catalyzed CO_2_ removal gave the product **12**. The HWE coupling of **12** with diethyl phosphonoacetonitrile **49** gave 3,7-dimethylocta-2,6-dienenitrile **71**. The open chain nitrile **71** is then cyclized to α-cyclocitronitrile **72** with the concentrated sulfuric acid in nitromethane. The ring closure mainly gave the nitrile with the double bond in α-position. In the work-up procedure, it is proved to be important to keep the condition acidic to avoid a double bond shift to the β-position. α-Cyclocitronitrile **72** which upon DIBAL-H reduction and subsequent base treatment is converted into β-cyclocitral **51**. All starting materials used in this scheme namely acetic acid, acetonitrile and acetone are commercially available in any ^13^C-enriched form. β-Cyclocitral **51** is very efficiently converted into β-ionylidene acetaldehyde **46** *via* the HWE coupling with (4-diethylphosphono)-3-methylbut-2-enenitrile **50** (Scheme 11) and subsequent DIBAL-H reduction of nitrile function (Scheme 12). Further coupling of aldehyde **46** with C_5_-phosphonate **50** and subsequent DIBAL-H reduction of nitrile function of retinonitrile **48** (Scheme 10) afforded retinal **1.**

The isotopomers of (4-diethylphosphono)-3-methylbut-2-enenitrile **50** are obtained *via* the reactions mentioned in Scheme 12. The necessary starting reagent is isotopically labeled 1-chloroacetone which is obtained from isotopically labeled ethyl 3-oxobutanoate **70**. The anion of ethyl 3-oxobutanoate **70** is treated with SO_2_Cl_2_ to give the ethyl 2-chloro-3-oxobutanoate. Acid catalyzed CO_2_ expulsion gave 1-chloroacetone in any isotopically enriched form. Diethyl phosphonoacetonitrile **49** can also be obtained in any isotopomeric form by treating the anion of ^13^C-enriched acetonitrile with diethyl chlorophosphate.

The LiAlH_4_ reduction of ethyl 3-methylbut-2-enoate **62** to the required allylic alcohol (before bromination step) is obtained in low yield. An alternative high yield method is the reaction of acetone **8** with diethyl phosphonoacetonitrile **49** to give 3-methylbut-2-enenitrile. Subsequent DIBAL-H reduction of nitrile function afforded an aldehyde and reduction of the aldehyde with NaBH_4_ gave 3-methylbut-2-en-1-ol which upon bromination afforded 1-bromo-3-methylbut-2-ene **58**.

It has been observed that the anion of C_5_-phosphonate **50** (prepared in such a way that no base is present in the reaction mixture) reacted with β-cyclocitral **51** at room temperature to give only all-*E* β-ionylidene acetaldehyde **46**. The extension of product **46** up to retinal **1** under the same conditions gave only the pure *E*-isomer intermediates (Scheme 12) [93]. However, the stereochemistry depends on the substitution pattern of the phosphonate and the aldehyde and the conditions of the reaction.

The possibility of an *E*/*Z* isomeric mixture in the reaction is due to the delocalized allylic anion structure of **50**. In case of the anion of **50** above -20 °C the isomerization is rapid leading within experimental error to only all-*E* structure. The corresponding allylic anion in which the nitrile function is substituted by an ester function also showed rapid isomerization above -20 °C but the thermodynamic equilibrium is composed of about *E*/*Z* (1:1) isomeric anion mixtures [94]. *E*-Selectivity is exclusive due to thermodynamic preference of linear nitrile function in phosphonate derivative **50** compared to the triangular ester function of the corresponding phosphonate derivative.

It has been reported that a HWE reaction of the anion of ethyl diethylphosphonoacetate with aldehyde gives mainly *E*-product, where as the ethyl diphenylphosphonoacetate and the bis(trifluoroethyl)phosphonate ester give only *Z* product [95,96]. It has been discussed that the formation of the more stable *trans*-olefin is reached *via* the threo-adduct whereas the better leaving diphenylphosphonate group reacts to give the *cis*-olefin *via* the erythro-adduct [97].

The HWE coupling of β-ionylidene acetaldehyde **46** with either the anion of 4-[bis(trifluoro)ethylphosphono]-3-methylbut-2-enenitrile or 4-(diphenylphosphono)-3-methylbut-2-enenitrile gave in good yield 11*Z*-retinal mixed with the all-*E* form [93,98,99]. The fact is that the presence of more electron withdrawing phosphonate in the allylic nitrile didn’t give complete *Z* formation as in the case of ethyl diethylphosphonoacetate. This can be explained by the greater stability of the allylic anion which leads to reversibility of the erythro-adduct to the starting reagents. Under these conditions also threo-adduct can form which gives *trans* isomer by the elimination of diphenyl phosphate salt. In the mean time *via* this method 3,4-didehydro-11*Z*-retinal and 7,8-dihydro-11*Z*-retinal have been prepared [100,101].

In order to test if a better leaving group would lead to the formation of 11*Z*-retinal only, the Arbuzov reaction of bis(4-nitrophenyl)methylphosphite with 4-chloro-3-methylbut-2-enenitrile was attempted, however even at very high temeperature no bis(4-nitrophenyl)methylphosphonate is formed. In order to test the possibilities for pure 11*Z*-retinal formation in a HWE reaction the required phosphonate with better leaving groups have to be prepared in another way than by Arbuzov reaction.

## 3. Isotope Enriched Chemically Modified Retinals

### 3.1. Preparation of (11Z)-3,4-didehydroretinal, (3R)-(11Z)-3-hydroxyretinal and (4R)-(11Z)-4-hydroxyretinal

**Scheme 18:**
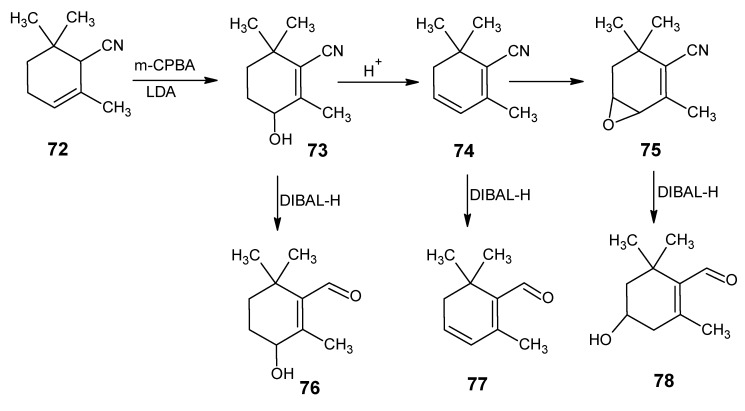
The conversion of α-cyclocitronitrile **72** into (4*RS*)-4-hydroxy-β-cyclocitral **76,** safranal **77** and (3*RS*)-3-hydroxy-β-cyclocitral **78**.

Besides 11-*Z* retinal in some animals (11*Z*)-3,4-didehydroretinal, (3*R*)-(11*Z*)-3-hydroxyretinal and (4*R*)-(11*Z*)-4-hydroxyretinal are the chromophores of the visual pigment. The reactions depicted in the synthetic route in Scheme 18 are used for the preparation of above mentioned retinals [13]. α-Cyclocitronitrile **72** (Scheme 17 and Scheme 18) is used as the starting material in which the double bond can easily be converted into an epoxide ring and subsequent treatment with a base gives (*R/S*)-4-hydroxy unsaturated nitrile **73**. In the presence of acid a water molecule is eliminated to give the nitrile **74**.

Treatment of **74** with m-chloroperbenzoic acid afforded the product **75** *via* the epoxidation of γ,δ-double bond. DIBAL-H reduction of products **73**, **74** and **75** afforded aldehydes (4*RS*)-4-hydroxy-β-cyclocitral **76**, safranal **77** and (3RS)-3-hydroxy-β-cyclocitral **78**, respectively. The required (4*R*)-(11*Z*)-4-hydroxyretinal, (11*Z*)-3,4-didehydroretinal and (3*R*)-(11*Z*)-3-hydroxyretinal, respectively are obtained by the HWE coupling of the aldehydes **76**, **77** and **78** in Scheme 18 with the anion of the phosphonate **50** and subsequent DIBAL-H reduction of the corresponding nitrile function [13].

A serious drawback in the synthetic routes shown in Scheme 17 and Scheme 18 used for the preparation of retinals and chemically modified retinals with the modification in the six membered ring are linear, as a result lower yield of the required β-cyclocitral **51** and even lower yields of (4*RS*)-4-hydroxy-β-cyclocitral **76**, safranal **77** and (3*RS*)-3-hydroxy-β-cyclocitral **78** are obtained.

**Scheme 19:**
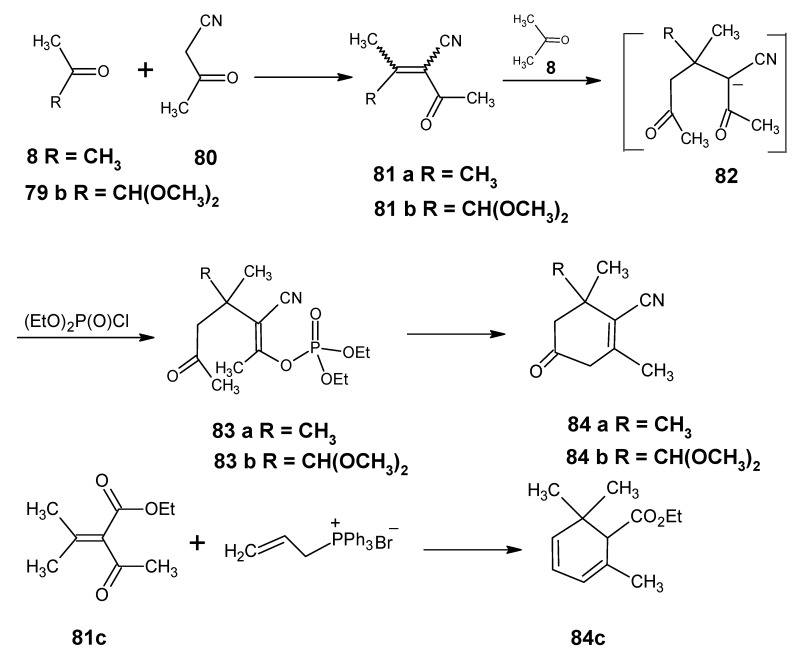
Preparation of β-cyclocitral derivatives *via* Knoevenagel condensation.

A more convergent method to obtain the required β-cyclocitral derivatives in high yield is indicated in Scheme 19. The Knoevenagel reaction of 1-cyanoacetone **80** with acetone **8** and 1,1-dimethoxyacetone **79b** has been reported [102]. Products *E*/*Z* 4-methyl-3-cyanopent-3-ene-2-one **81a** and E/Z 5,5-dimethoxy-4-methyl-3-cyanopent-3-ene-2-one **81b** are obtained in high yield in a one-step procedure. The nucleophilic attack on these highly poor alkenes is expected to take place on carbon 4. In this case anion of acetone **8** is prepared from LDA at -90 °C in THF. This is expected to lead to the anion **82**. Subsequent reaction with diethyl chlorophosphate should result in the cyano enol phosphate **83** which is expected to undergo a ring closure to give 5-oxo-β-cyclocitronitrile **84a** or dimethoxy derivative of 5-oxo-β-cyclocitronitrile **84b**.

The reaction of **81c** (the ester analogue of **81a**) with the anion of allyltriphenylphosphonium bromide to obtain the citral derivative **84c** in Scheme 19 has been reported [103]. This means that the reactions in Scheme 19 will lead to a very convenient method to obtain retinals with the ^13^C-enrichment in the six-membered ring or the chemically modified retinals with the modification in the six membered ring. The 16,17-dimethoxyretinals also open the possibility to obtain retinals that differ in isotope composition on carbons 16 and 17 in pure enantiomeric form. Previously, the Knoevenagel reaction has been used in the field of vitamin A for the conversion of β-ionone **17** into β-ionylidene acetonitrile **85** [104].

### 3.2. Preparation of 10-methylretinal, 10-methylthioretinal, 10-iodoretinal and 19-fluororetinal via β-ionylidene acetonitrile ***85***

**Scheme 20:**
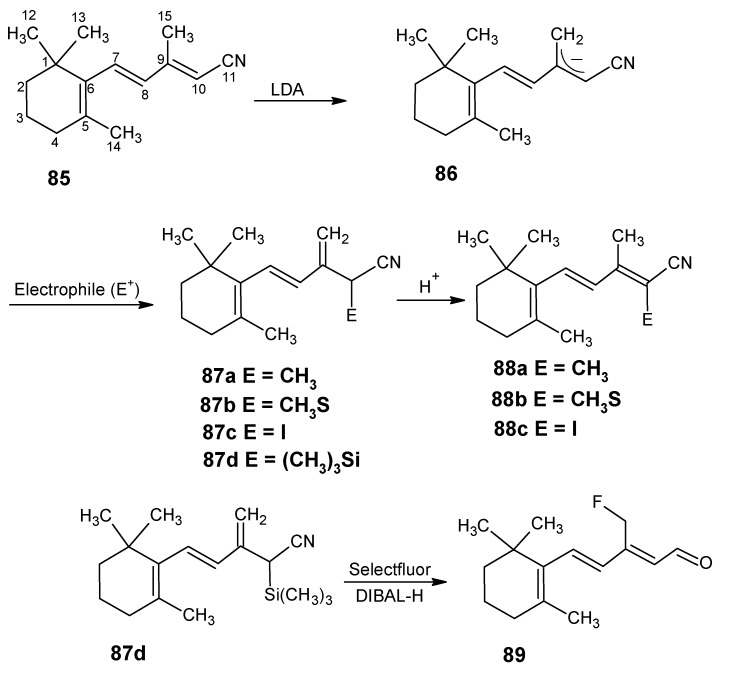
The preparation of chemically modified retinoids by using the reactivity of conjugated nitriles.

In Scheme 20 a synthetic route is depicted to obtain the chemically modified retinoids by using the reactivity of conjugated nitriles. β-Ionylidene acetonitrile **85** is treated with LDA in THF resulting in the allylic anion **86** by deprotonation of methyl group at postion 15. Treatment of **86** with the electrophilic reagent CH_3_I gave **87a** with the methyl group at position 10. In the presence of an acid allylic shift occurred to give 10-methyl-β-ionylidene acetonitrile **88a** [105].

Similarly, the allylic anion **86** with methyl thiocyanate, iodine and trimethylsilyl chloride gave 10-methylthio-β-ionylidene acetonitrile **88b**, 10-iodo-β-ionylidene acetonitrile **88c** and trimethylsilyl derivative **87d**, respectively. The later is treated with selectfluor^(R)^, followed by DIBAL-H reduction to obtain 15-fluoro-β-ionylidene acetaldehyde **89** [106]. The nucleophilic attack on the allylic trimethylsilanes is a general reaction, which means that a large number of 15-substituted-β-ionylidene acetonitriles will be accessible *via* this method. DIBAL-H reduction of these commercially modified β-ionylidene acetonitriles will give the corresponding β-ionylidene acetaldehydes, which can easily be converted into the corresponding retinonitriles.

Similarly, using building blocks **88a**, **88b** and **88c** 10-methyl-, 10-methylthio-, and 10-iodoretinonitriles are prepared, respectively [105]. It is clear that using the acidity of methyl groups in conjugated nitriles retinals with chemical modification or combinations of modifications at positions 10, 14, 19 and 20 are accessible. The required conjugated nitriles are accessible in any isotopically enriched form which means that chemically modified retinals are accessible *via* this method. Retinals modified with alkyl groups are available in any isotopically enriched form *via* above described method because the simple alkyl groups are available in any isotopically enriched form.

### 3.3.Preparation of 11-methylretinal and 12-methylretinal via β-ionyl triphenylphosphonium bromide 90

**Scheme 21:**
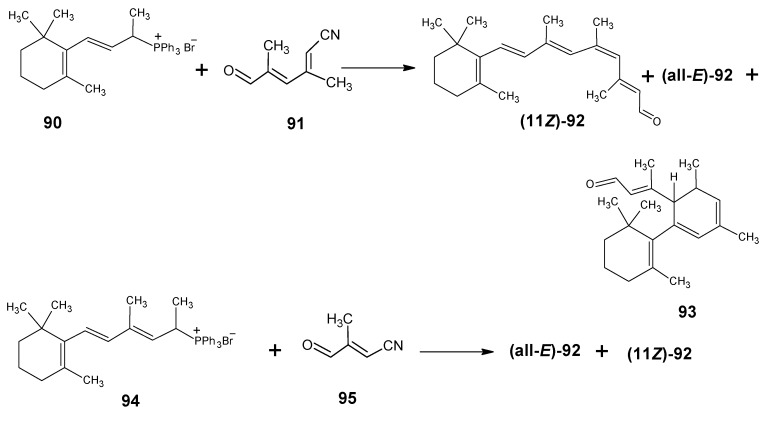
The preparation of 11-methylretinal **92** *via* a HWE reaction.

The preparation of 11-methylretinal is carried out *via* the reactions depicted in Scheme 21. β-Ionyl triphenylphosphonium bromide **90** is refluxed in 1,2-epoxybutane in the presence of 3,5-dimethyl-6-oxohexa-2,4-dienenitrile **91** [107]. The later is prepared *via* the HWE reaction of 1,1-dimethoxyacetone **79b** and the anion of the phosphonate **50** (Scheme 11) followed by the acetal deprotection to obtain the aldehyde **91**. After DIBAL-H reduction (all-*E*)-11-methylretinal and (9*Z*)-11-methylretinal are obtained. The HPLC separation also gave a new product **93** in which the anion of **90** is reacted in a 1,4-addition to the conjugated aldehyde **91**. After proton shift an internal Wittig reaction took place leading to the corresponding nitrile **93**.

The 1,4-conjugated Wittig reactions that lead to **93** are known [108,109,110,111,112]. The ratio (all-*E*)-**92**:(9*Z*)-**92**:**93** = 1:1:2 of the obtained products showed that (all-*E*)-**92** and (9*Z*)-**92** are formed in a 1:1 ratio that is in agreement with leaving group facility in triphenylphosphine oxide. This has been discussed in the industrial syntheses. When triphenylphosphonium bromide **94** is treated with the conjugated aldehyde **95** in refluxing 1,2-epoxybutane only (all-*E*)-**92** and (11*Z*)**-92** are formed just as in the BASF technical synthesis. Product **94** is prepared by methylation *via* Grignard reagent on β-ionylidene acetaldehyde **46** and subsequent triphenylphosphonium salt formation. Building block **95** has been prepared *via* coupling of 1,1-dimethoxyacetone **79b** and the anion of diethyl phosphonoacetonitrile **49**. This improved synthesis of 11*Z*- and all-*E* 11-methylretinal **92** is depicted in lower line of Scheme 21. In this reaction no side product **93** is formed. It is clear that the 9-methyl group in the reagent **94** prevents the formation of products *via* 1,4-Wittig reaction. In the BASF synthesis the presence of 9-CH_3_ also prevents the formation of side products.

**Scheme 22:**
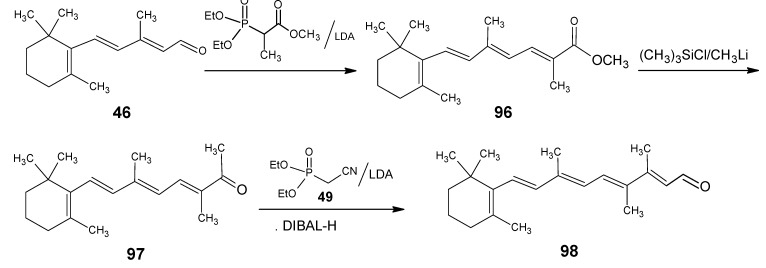
Preparation of 12-methylretinal **98** starting from β-ionylidene acetaldehyde **46**.

For the formation of 12-methylretinal the reactions in the Scheme 22 are used [105]. β-Ionylidene acetaldehyde **46** is coupled with the anion of methyl 2-(diethylphosphono)propionate to give the corresponding ester **96**. Treatment of the ester **96** with methyl lithium and trimethylsilyl chloride gave the methyl ketone **97** which is easily extended to 12-methyl retinal **98**. Methyl 2-(diethylphosphono)propionate is easily obtained by a Hell-Volhardt-Zelinsky reaction on propionic acid and followed by treatment with triethylphosphite under Arbuzov condition. All carboxylic acids will give in the same way methyl 2-(diethylphosophono)carboxylates. This means that a whole series of retinals with different alkyl groups at position 12 is accessible *via* this method.

### 3.4. Preparation of 9-demethyl-9-haloretinals and 13-demethyl-13-haloretinals

**Scheme 23:**
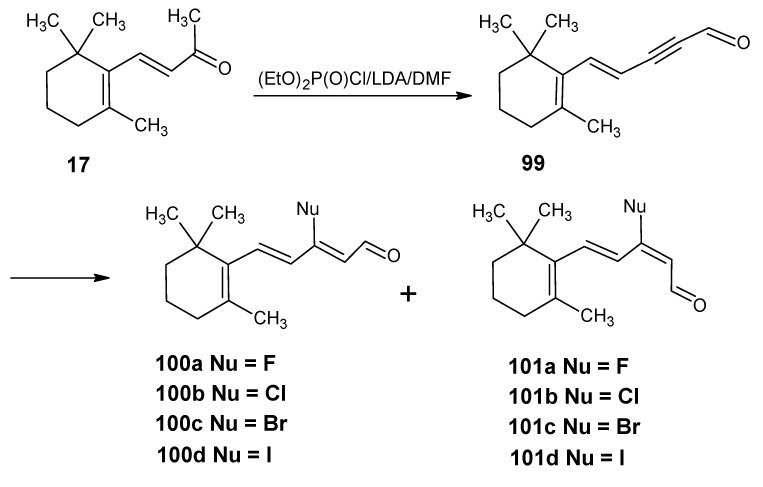
Preparation of (9*Z*)- 9-halo-β-ionylidene acetaldehyde **100a**, **100b**, **100c**, **100d** and their all-*E* isomers **101a**, **101b**, **101c**, **101d**.

The synthetic route depicted in Scheme 23 shows that β-ionone **17** can be easily converted into (9*Z* **)-** and all-*E* 9-fluoro-, 9-chloro-, 9-bromo- and 9-iodo-β-ionylidene acetaldehyde (**100** and **101**) [93]. The anion of β-ionone **17** is obtained by the reaction of β-ionone **17** with LDA by the removal of proton from the methyl ketone group. This anion is reacted with diethyl chlorophosphate to form the enol ether phosphate. The addition of a second equivalent of LDA gave acetylide anion by the elimination of phosphate group. The addition of one equivalent of dimethylformamide afforded a one-pot formation of the acetylene acetaldehyde **99**. ^13^C-Dimethylformamide is commercially available; this means product **99** is accessible in any isotopically enriched form.

Treatment of product **99** with LiCl, LiBr, and LiI in acetic acid at 70 °C gave the complete conversion into (9*Z*)-9-halo-β-ionylidene acetaldehydes **100b**, **100c**, **100d** and their all-*E* isomers, respectively. The mixtures of two *E*/*Z* isomers could easily be separated by chromatography. Treatment of product **99** with commercially available tetrabutylammonium dihydrogentriflouride in 1,2-dichloroethane at 80 ^O^C gave the two 9-fluoroderivatives **100a** and **100b** together with unconverted starting material **99** and product **101a** and **101b**. The two fluorides could easily be separated in pure form by chromatography. The chlorides **100b** and **101b** are the result of the F^-^ ion induced Cl^-^ ion release from the 1,2-dichloroethane. The derivatives **100a**, **100b**, **100c**, **100d**, **101a**, **101b**, **101c** and **101d** are converted into the 9-demethyl-9-haloretinals *via* a HWE reaction with the anion of the phosphonate **50**. In this way the 9-fluoro-, 9-chloro-, 9-bromo-, and 9-iodoretinals are accessible in the all-*E*, 9*Z*- and 11*Z*-isomeric form. In a similar way it is to be expected that the C_18_ ketone **47** in Scheme 10 can be converted into the geometric isomers of the 13-demethyl-13-haloretinals.

## 4. α-Retinals *via* α-Ionone

### 4.1. 9-Demethyl-9-halo-α-retinals, 9-substituted α-retinals, 9-demethyl α-retinal, 19,19-ethano-α-retinal, 19,19-dimethyl α-retinal and 12- and 14-halo substituted α-retinals

**Scheme 24:**
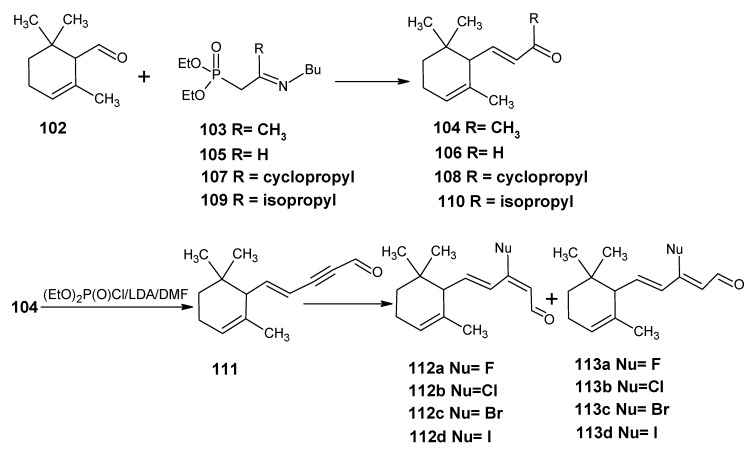
Preparation of α-ionylidene acetaldehydes (halogen substituted at position 9) **112** and **113** starting from α-cyclocitral **102**. Substituent R in phosphonate is varied to obtain the chemically modified α-ionones **106**, **108** and **110**.

In Scheme 24 the synthetic route is shown to prepare 4,5-didehydro-9-demethyl-9-halo-5,6-dihydroretinals in the pure all-*E*, 9*Z*- and 11*Z*-isomer *via* the 9-demethyl-9-halo-α-ionylidene acetaldehyde (**112** and **113**) [113]. α-Cyclocitronitrile **72** (Scheme 18) is treated with DIBAL-H to give pure α-cyclocitral **102**. α-Ionone **104** is obtained in pure form by the treatment of α-cyclocitral **102** with the anion of diethyl [2-(butylimino)propyl]phosphonate **103**. Usually a simple aldol condensation of α-cyclocitral **102** with acetone **8** gave the β-ionone **17** *via* a base catalysed conversion but in this case pure α-ionone **104** is obtained. The 9-methyl-9-halo-α-ionylidene acetaldehydes **112a**, **112b**, **112c**, **112d** and **113a**, **113b**, **1^13^C**, **113d** are obtained *via* the acetylene acetaldehyde **111** in the reaction sequence similar to the reactions described for β-ionylidene series in Scheme 23. The *E/Z*-isomers of **112** and **113** could be easily obtained in pure isomeric form. A final coupling with the anion of **50** afforded the 9-demethyl-9-halo-α-retinals in pure all-*E*, 9*Z*- and 11*Z*-isomeric form.

α-Cyclocitral **102** is converted into α-ionone **104** *via* the HWE reaction of the anion of diethyl [2-(butylimino)propyl]phosphonate **103** (Scheme 24). The substituent R in phosphonate derivative is varied to obtain phosphonate derivatives **105,107** and **109** which gave α-ionone derivatives without a keto methyl group **106**, with a cyclopropyl group **108** and an isopropyl group **110**, respectively. For HWE reagents (**103**, **105**, **107** and **109**) imines of the carbonyl compound in question are prepared. The anion of the imine (prepared by the addition of an equivalent of LDA) is treated with one equivalent of diethyl chlorophosphate to obtain a phosphonate derivative. By the additional equivalent of LDA to the phosphonate derivative phosphonate carbanion is generated *in situ*, final nucleophilic addition of the carbanion onto the α-cyclocitral **102** gave the corresponding α-ionone derivative.

Interestingly, such a process did not work in the Peterson olefination strategy [114]. Treatment of the anion of ketimine with trimethylsilyl chloride leads to N-silylation whereas in the preparation of phosphonate derivative only C-phosphonylation takes place. It is clear that the Peterson reagent *N*-[2-(trimethylsilyl)ethylidene]methamine used in Scheme 10 is an exception where the presence of sterically bulky group forces the silylation on the carbon atom.

The α-ionones that are prepared *via* the synthetic route in Scheme 24 did not show any trace of the corresponding β-ionones. These α-ionones could easily be converted into the corresponding 9-substituted α-retinals in all-*E*, 9*Z*-, 11*Z*-isomeric form. It is clear that besides the prepared α-retinal a whole series of 9-substituted α-retinals will be accessible *via* the reactions depicted in Scheme 24. In this way besides 9-substituted α-retinals also 9-demethyl-α-retinal, 19,19-ethanoretinal and 19,19-dimethyl-α-retinal *via* phosphonate derivatives **105**, **107** and **109**, respectively are prepared [106,113]. For the preparation of phosphonate derivatives **107** and **109** methyl cyclopropyl ketone and methyl isopropyl ketone, respectively are used. These building blocks are not commercially available in stable isotopically labeled form. However, they can be prepared by using commercially available isotope enriched starting materials *via* simple reactions mentioned in the schemes.

The α-retinals discussed in this paper are accessible in any isotope enriched form. The corresponding β-ionone derivatives can easily be prepared in pure form *via* the similar method described in Scheme 17 starting from β-cyclocitral **51**. The β-ionones so prepared can easily be converted into the corresponding retinals with different substituents at the position 9 in all-*E*, 9*Z*- and 11*Z*-isomeric form. But in the case of the base catalyzed aldol condensation of β-cyclocitral **51** with methyl isopropyl ketone and methyl cyclopropyl ketone mixtures of the α- and β-ionone derivatives are obtained which are very difficult to separate in pure form. It is clear that the strategy discussed so far gives an access to many substituted retinals and their α-isomers with substituents on various positions and full access to any stable isotopically enriched form.

**Scheme 25:**
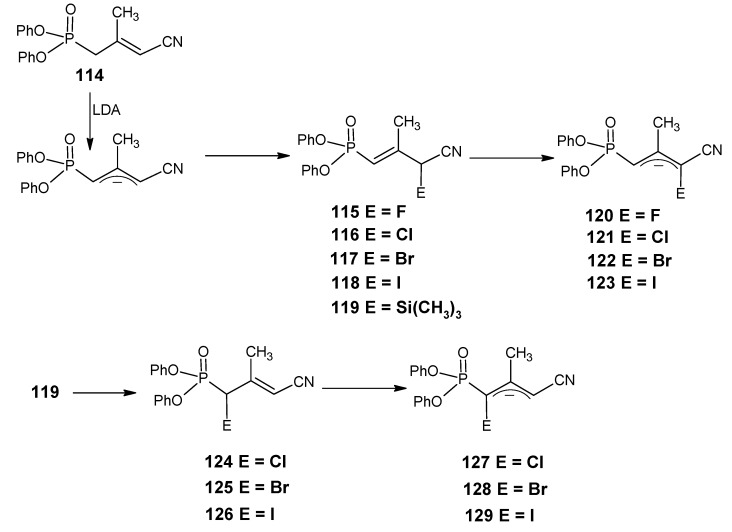
The chemical modification of 4-(diphenylphosphono)-3-methylbut-2-enenitrile **114** to prepare 12- and 14-halo substituted retinals and α-retinals in the all-*E*, and 11*Z*-isomeric form.

Another approach to obtain modified retinals is *via* the chemical modification of the 4-(diethylphosphono)-3-methylbut-2-enenitrile **50** and its diphenyl homologue 4-(diphenylphosphono)-3-methylbut-2-enenitrile **114**. The chemical modification of phosphonate **114** is mentioned in Scheme 25. Treatment of phosphonate derivative **114** with base gave phosphonate carbanion which reacted with electrophilic reagents such as selectfluor, NCS, NBS, NIS and trimethylsilylchloride to give the corresponding products **115**-**119** with the substitution exclusively at position 2 with respect to the nitrile function [106]. The phosphonate group directs the incoming electrophile towards the γ-position with respect to itself [115]. Similarly, nitrile function directs the incoming electrophile towards the α-position with respect to itself.

However, electrophilic substitution in the phosphonate **114** is achieved at position 4 by using the trimethylsilyl as a temporary leaving group. The silylderivative **119** is treated with NCS, NBS and NIS to give the products **124**-**126** with halogen substitution at position 4. However, the silylderivative **119** didn’t react with selectfluor [106]. The anion of the corresponding phosphonate derivative **120**, **121**, **122** and **123** is coupled with β-ionylidene acetaldehyde **46** to give (11*E*/11*Z*)-14-haloretinals. Similarly, the coupling of the anion of the corresponding phosphonate derivatives **127**, **128** and **129** with β-ionylidene acetaldehyde **46** gave (11*E*/11*Z*)-12-haloretinals.

Similarly, phosphonate **50** is converted into substituted phosphonate reagents by the substitution reactions described above. These reagents under the right conditions led to pure retinals and α-retinals with the newly formed carbon 11-carbon 12 double bond in the *E*-configuration. Coupling of the HWE reagents depicted in Scheme 24 with either β-cyclocitral **51** or α-cyclocitral **102** led to β- and α-ionylidene acetaldehydes, respectively with substitution at position 8 or 10 (Scheme 20). Furthermore, treatment of these chemically modified α- and β-ionylidene acetaldehydes again with the reagents in Scheme 25 will result in retinals and their α-isomer chemically modified at positions 8, 10, 12 and 14 and all possible combination of modifications at these positions. All these novel systems are also accessible in any stable ^13^C labeled form.

## 5. Nor-Retinals

### 5.1. 16,17,18-Trinor-retinal, 16,17-dinor-retinal and 16-nor-retinal

**Scheme 26:**
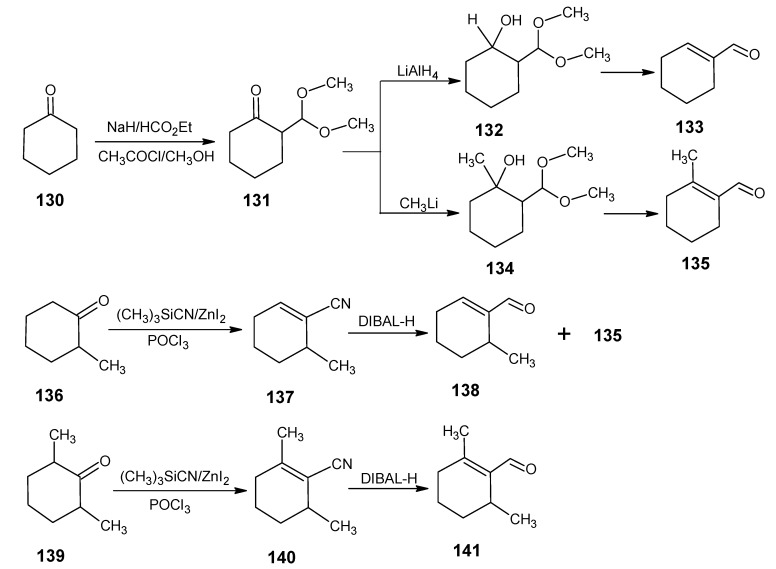
Conversion of cyclohexanone **130** into β-cyclocitral derivatives.

The synthetic routes depicted in Scheme 26 show that the β-cyclocitral derivatives are prepared starting from cyclohexanone derivatives. Cyclohexanone **130** is formylated with ethyl formate in the presence of base *via* the reactions in Scheme 26 [116].

The formylated cyclohexane derivative is treated with acetyl chloride and subsequently treated with methanol in the presence of acid to obtain the corresponding acetal **131**. Upon treatment with LiAlH_4_ the carbonyl function is reduced to alcohol **132**, the acid catalyzed deprotection of acetal function and elimination of a water molecule afforded cyclohex-1-en-1-al **133**.

Similarly, reaction of the product **131** with methyl lithium followed by an acid catalyzed deprotection of acetal function and the elimination of a water molecule afforded 2-methylcyclohex-1-en-1-al **135**. The chain extension on these products **133** and **135** are carried out by the reaction of each with phosphonate **50** (Scheme 11) twice to afford 16,17,18-trinor-retinal and 16,17-dinor-retinal, respectively.

2-Methylcyclohexanone **136** is treated with trimethylsilyl cyanide and subsequent removal of water gave 6-methylcyclohex-1-en-nitrile **137** (mixed with other isomer). After DIBAL-H reduction of the product **137** 6-methylcyclohex-1-en-1-al **138** is easily separated from the isomeric product **135**. Similarly, starting from 2,6-dimethylcyclohexanone **139** 2,6-dimethylcyclohex-1-en-1-al **141** is obtained. These two aldehydes **138** and **141** are easily converted into 16,17-dinor-retinal and 16-nor-retinal, respectively.

**Scheme 27:**
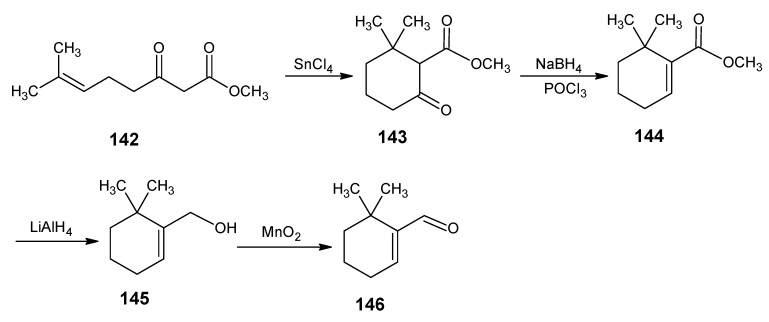
Preparation of 6,6-dimethylcyclohex-1-en-1-al **146** starting from methyl 7-methyl-3-oxo-6-octenoate **142**.

In Scheme 27 it is shown that 6,6-dimethylcyclohex-1-en-1-al **146** is prepared starting from methyl 7-methyl-3-oxo-oct-6-enoate **142** [117]. The keto ester **142** upon treatment with SnCl_4_ afforded cyclohexanone ester **143** followed by NaBH_4_ reduction and the removal of a water molecule to obtain cyclohexene ester **144**. LiAlH_4_ reduction and subsequent MnO_2_ oxidation of the product **144** gave β-cyclocitral derivative **146**. The aldehyde **146** *via* above mentioned procedure is converted into 5-demethylretinal.

**Scheme 28:**
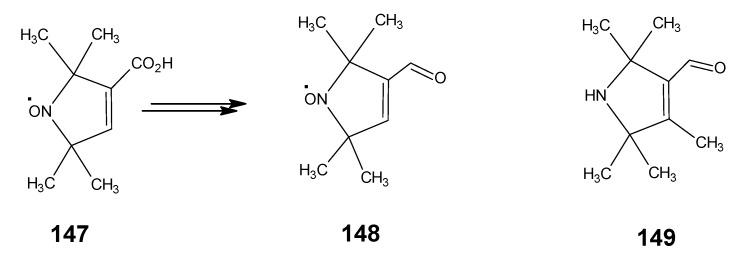
Preparation of nitroxide aldehyde **148** and the corresponding pyrroline aldehyde **149**.

In Scheme 28 it is depicted that the commercial nitroxide product **147** can be converted into the *N*-methoxy-*N*-methyl amide derivative and subsequent DIBAL-H reduction into the spin label **148** [118]. The cyclocitral derivative **149** could be prepared in a similar way. Both products **148** and **149** could be converted into the corresponding retinals by adjusting the HWE reagent **50** (Scheme 11) into the corresponding esters. After the HWE coupling the corresponding conjugated esters are obtained which are converted into the corresponding *N*-methoxy-*N*-methyl amide derivatives. DIBAL-H reduction of these amides gave the corresponding aldehydes. However, DIBAL-H reduction of the conjugated nitriles obtained *via* the reaction of phosphonate **50** didn’t give useful results in the case of the nitroxyl and the corresponding amide containing system.

## 6. Bridged and Demethyl Retinals

### 6.1. DL-8,16-Methanoretinal, 8,18-methanoretinal, (R)-5-demethyl-8,16-methanoretinal, 1,5-didemethyl-8,16-methanoretinal, 1,1-didemethyl-8,18-methanoretinal, 1,1-didemethyl-18-didehydro-8,18-methanoretinal

**Scheme 29:**
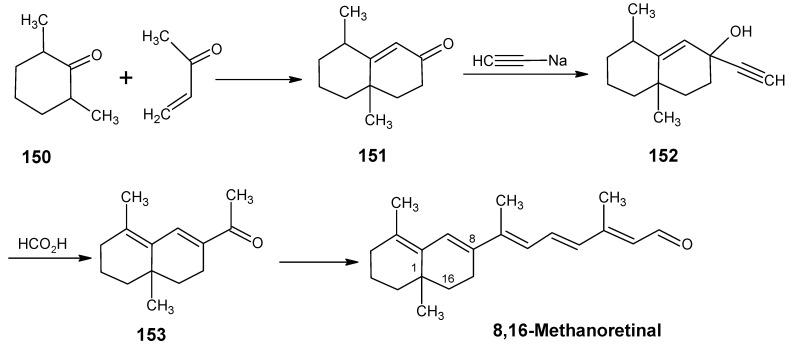
Preparation of racemic β-ionone derivative **153** starting from 2,6-dimethylcyclohexanone **150**.

**Scheme 30:**
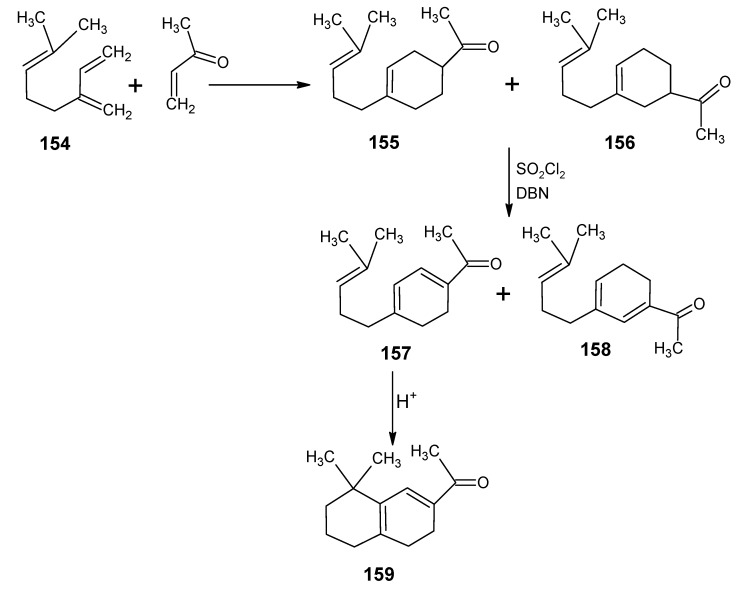
Preparation of bicyclic β-ionone **159** starting from myrcene **154**.

The starting β-ionone derivatives **153** and **159** needed to prepare DL-8,16-methanoretinal and 8,18-methanoretinal, respectively are obtained *via* the synthetic routes mentioned in Scheme 29 and Scheme 30 [119]. 2,6-Dimethylcyclohexanone **150** is converted into racemic bicyclic enone **151** *via* a Robinson annulation reaction with methylvinyl ketone. Upon treatment with the acetylide anion the tertiary propargylic alcohol **152** is obtained. Treatment of the alcohol **152** with formic acid gave a conjugated acetylene derivative (not shown in the Scheme 29) by the removal of water molecule and subsequent addition of water on the triple bond in the presence of acid afforded the racemic β-ionone derivative **153**.

The commercially available myrcene **154** underwent a Diels-Alder reaction with methylvinyl ketone to give a mixture of two Diels-Alder products **155** and **156**. Treatment of these products with SO_2_Cl_2_ chlorinated the tertiary carbon next to the carbonyl function. Elimination of HCl *via* the reaction with DBN gave cyclohexadienones **157** and **158**. These products are separated easily. Treatment of product **157** with sulfuric acid afforded the bicyclic β-ionone **159** which is further converted into the corresponding 8,18-methanoretinal.

**Scheme 31:**
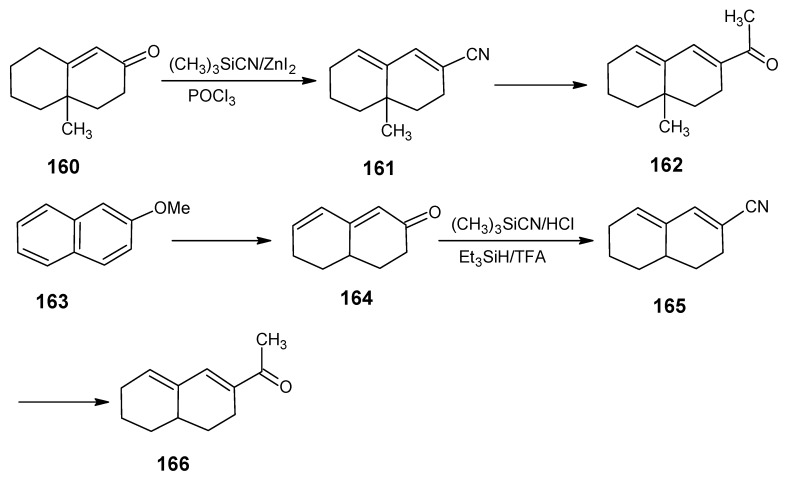
Preparation of β-ionone derivatives **162** and **166** from starting from (*R*)-bicyclic ketone **160** and 2-methoxynaphthalene **163**, respectively.

In Scheme 31 the synthetic route is depicted to show that the commercially available (*R*)-bicyclic ketone **160** is converted into a β-ionone derivative **162** [120]. Reaction with trimethylsilyl cyanide in the presence of zinc iodide and the elimination of a molecule of water gave the conjugated nitrile **161**. Treatment of the later with methyl lithium afforded the (*R*) form of the β-ionone derivative **162**. That can be further converted into (*R*)-5-demethyl-8,16-methanoretinal. Also, the corresponding S form of bicyclic ketone is commercially available. Similarly, following the reactions described above (S)-5-demethyl 8,16-methanoretinal is obtained *via* the intermediate (*S*) form of the β-ionone derivative. In the same paper racemic 5-demethyl-8,16-methanoretinal with deuterium incorporation at positions 5 and 7 have been described starting from 2-methylcyclohexanone (Scheme 29).

In the lower line of Scheme 31 the synthetic route is given to show that 2-methoxynaphthalene **163** is converted into the β-ionone derivative **166** [121]. Birch reduction of **163** gave 1,4,5,8-tetrahydro-2-methoxynaphthalene which upon treatment with acid afforded the bicyclic ketone **164**. The product **164** is treated with trimethylsilyl cyanide and subsequent HCl treatment gave the cyanohydrin *via* desilylation. The later product is treated with triethylsilane and trifluoroacetic acid. The carbinol is converted into the carbenium ion, which is reduced with triethylsilane into the bicyclic nitrile **165**. Treatment of the product **165** with methyl lithium afforded the β-ionone derivative **166**. The product **166** is converted into (RS)-1,5-didemethyl-8,16-methanoretinal.

**Scheme 32:**
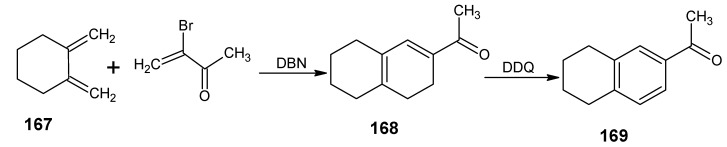
Preparation of β-ionone derivative **168** and its aromatic counterpart **169**.

In Scheme 32 the synthetic route is shown for the preparation of β-ionone derivatives **168** and **169**. Diels-Alder reaction of 1,2-bis(methylene)-cyclohexane **167** and 3-bromobut-3-en-2-one and subsequent HBr elimination afforded the β-ionone derivative **168**. Oxidation of the product **168** with DDQ gave the aromatic β-ionone derivative **169**. The β-ionone derivatives **168** and **169** could easily be converted into all-*E* 1,1-didemethyl-8,18-methanoretinal and 1,1-didemethyl-18-didehydro-8,18-methanoretinal, respectively [121].

### 6.2. 11,19-10,20-Dimethanoretinal, 10,20-methanoretinal, 13-demethyl-10,12-ethanoretinal, 13-demethyl-12,14-ethanoretinal, 13-demethyl-10,12-propanoretinal and 13-demethyl-12,14-propanoretinal

**Scheme 33:**
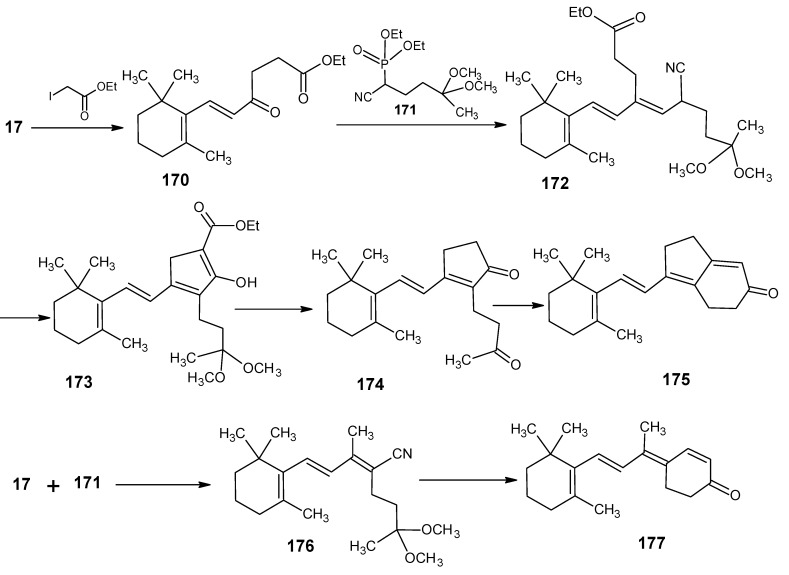
Reaction of β-ionone **17** leading to the C_20_ ketone **175** and C_19_ ketone **177**.

In Scheme 33 the synthetic route is depicted to show the conversion of β-ionone **17** into C_20_ ketone **175** and C_19_ ketone **177** [122]. β-Ionone **17** is alkylated with ethyl iodoacetate in the presence of a base to give a keto ester **170**. A HWE reaction of the keto ester **170** with the cyanophosphonate **171** afforded the conjugated nitrile **172**. Product **171** is easily accessible from the commercially available 5-chloropentan-2-one. Treatment of the product **172** with t-butanolate base afforded the product **173** which in the presence of the acid gave the diketone **174**. Aldol condensation of the product **174** afforded the locked enone system **175** which is converted into 11,19-10,20-dimethanoretinal. In the lower line of the Scheme 33 it is shown that a HWE coupling of β-ionone **17** and cyanophosphonate **171** afforded the conjugated nitrile **176**. Further DIBAL-H reduction followed by the acetal deprotection of the product **176** and subsequent aldol condensation led to a locked C_19_ ketone derivative **177**. The product **177** has been converted into 10,20-methanoretinal into the (10,13 all-*E*)-, (10*Z*, 13*E*)-, (10*E*, 13*Z*)- and (10*Z*, 13*Z*) -isomers which could be simply isolated in pure form.

**Scheme 34:**
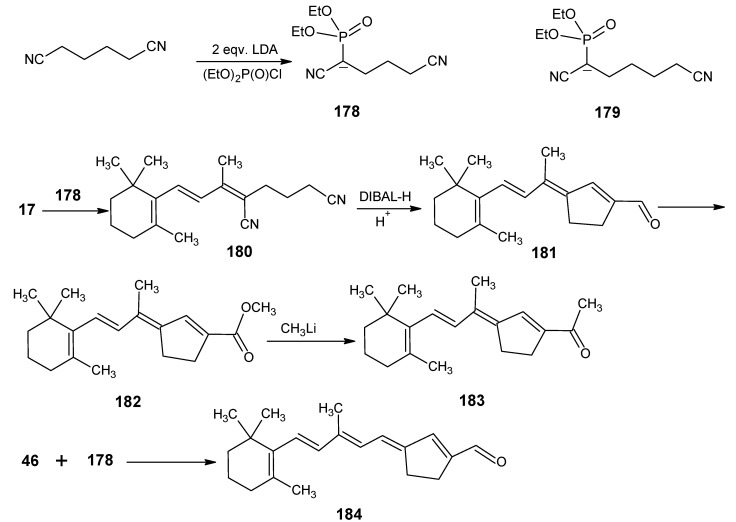
Nitrile phosphonates **178** and **179** which are used to prepare of 13-demethyl-10,12-ethanoretinal**181** and 13-demethyl-12,14-ethanoretinal **184** and their corresponding propano- derivatives.

In Scheme 34 the synthetic route is depicted to show the conversion of 1,4-dicyanobutane to the HWE reagent cyanophosphonate **178** by the reaction of 2 equivalents of LDA and one equivalent of diethyl chlorophosphate [123]. This reagent can be used to introduce ethano-bridge in the retinal structure. Similarly, the HWE reagent **179** can be prepared from 1,5-dicyanopentane. With the cyanophosphonate **179** propano-bridged retinals can be prepared.

The reaction of β-ionone **17** and cyanophosphonate **178** afforded the dinitrile **180**. DIBAL-H reduction and aldol condensation gave the product **181**. The product **181** can be easily converted into the ester **182**. The later upon treatment with methyl lithium in the presence of trimethylsilyl chloride gave the methyl ketone **183**. The later can easily be converted into 13-demethyl-10,12-ethanoretinal. Similarly, by the reaction of β-iononylidene acetaldehyde **46** and cyanophosphonate **178** 13-demethyl-12,14-ethanoretinal **184** is obtained. By repeating the reactions depicted in Scheme 34 with the reagent **179** 13-demethyl-10,12-propanoretinal and 13-demethyl-12,14-propanoretinal are accessible.

### 6.3. 13-Demethyl-10,14-thiaretinal and 11,14-bridged 13-demethyl retinals

**Scheme 35:**
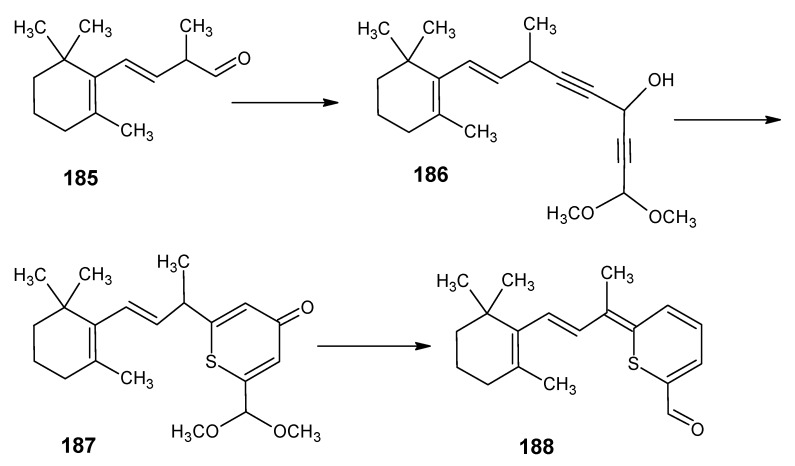
Preparation of (9*Z*)-13-demethyl-10,14-thiaretinal from α-C_14_ aldehyde **185** and its all-*E* isomer (not indicated in the scheme).

In Scheme 35 the synthetic route is depicted to show the conversion of the α-C_14_ aldehyde **185** (an isomer of the product **21** in Scheme 3) into the thiaretinal **188**. The product **185** is treated with dibromomethyltriphenyphosphorane to give dibromoethene derivative. Debromination by butyl lithium afforded the corresponding acetylene derivative. Treatment of the product with 4,4-dimethoxybut-2-yn-1-al gave the diacetylene alcohol **186** [124]. MnO_2_ oxidation of the product **186** led to a ketone which is treated with thiourea to give the thiopyranone **187**. Treatment of the product **187** with DIBAL-H and subsequent deprotection of the acetal function afforded 9*Z*-derivative **188** and its all-*E* isomer which could easily be separated.

**Scheme 36:**
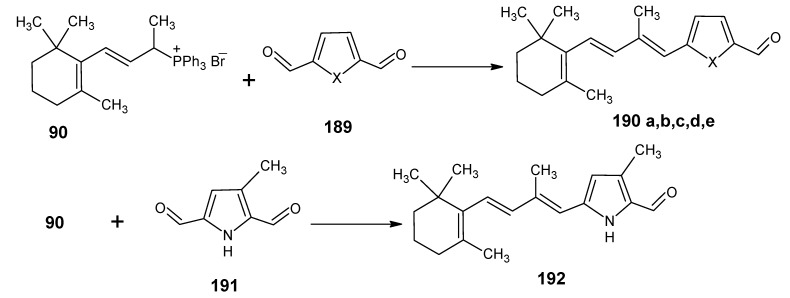
Preparation of 11,14-bridged 13-demethyl retinals **190 a-e** [**a** (X= NH), **b** (X= N-CH_3_), **c** (X= S), **d** (X= -CH=CH-), **e** (X= O)].

In Scheme 36 the synthetic route is depicted to show the reaction of β-ionyl triphenylphosphonium bromide **90** (Scheme 21) with aromatic dialdehydes **189 a-e** to obtain a mixture of all-*E* and 11*Z*-bridged retinals **190 a-e** [125]. The Wittig reaction is very selective by reacting only one aldehyde function of the dialdehydes **189** to give all-*E* and 11*Z*-products as it has been described in BASF technical syntheses. The Wittig reaction showed further selectivity with 3-methylpyrrole-2,5-dialdehyde **191** affording only 11*Z* and all-*E* mixture of 11,14-iminoretinal **192**.

### 6.4. 9-Demethyl retinal, 13-demethyl retinal and 9,13-didemethyl retinal

**Scheme 37:**
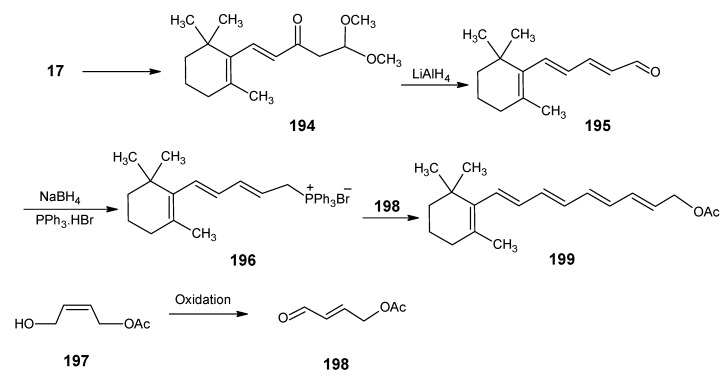
Preparation of 9,13-didemethyl retinyl acetate **199** *via* the HWE reaction of phosphonium bromide **196** and the conjugated acetate aldehyde **198**.

For the preparation of 9-demethyl-, 13-demethyl- and 9,13-didemethyl retinals the reactions in Scheme 6 for BASF preparation of vitamin A have been modified [126]. In Scheme 37 the synthetic route is depicted to show that β-ionone **17** is condensed with ethyl formate under basic conditions. The resulting aldehyde is converted into acetal **194**. Upon LiAlH_4_ reduction and deprotection afforded the conjugated aldehyde **195**. Mild NaBH_4_ reduction, followed by the reaction with HBr and phosphine gave phosphonium salt **196**. Commercial *cis*-butenediol was partially acetylated into the monoacetate **197**. Upon oxidation with pyridinium chlorochromate the conjugated aldehyde **198** is obtained.

The HWE reaction of phosphonium bromide **33** (Scheme 6) with the acetate aldehyde **198** gave a mixture of (11*Z*)- and all-*E* 13-demethyl retinyl acetate. The HWE reaction of phosphonium bromide **196** with the acetate aldehyde **35** (Scheme 6) gave a mixture of (11*Z*)- and all-*E* 9-demethyl retinyl acetate. The condensation of the phosphonium bromide **196** and the acetate aldehyde **198** afforded 9,13-didemethyl retinyl acetate **199** in 11*Z* and all-*E* forms. 9- and 13-Demethyl retinals in various deuterium enriched forms have been described [127].

## Conclusions

In this paper the contributions of the Leiden group to the site directed stable isotope enrichment in natural retinoids and chemically modified retinoids has been reviewed. It is clear that a modular approach to the Wittig chemistry on nitrile reagents provides access to any chemically modified retinoid isotopomer. Modifications in the synthetic schemes and easy modifications in the building blocks give simple access to any isotopomer of retinoids in a rational way. We dedicate this paper to the future investigators who will develop the field of stable isotope enriched retinoids, prepare the now accessible isotopomers and related compounds further in a fundamental way to explore the various aspects of the role of vitamin A in (human) life leading to an ever deeper understanding of the bio(chemistry) of retinoids.
